# Cumulative burden of 144 conditions, critical care hospitalisation and premature mortality across 26 adult cancers

**DOI:** 10.1038/s41467-023-37231-3

**Published:** 2023-03-17

**Authors:** Wai Hoong Chang, Richard D. Neal, Martin D. Forster, Alvina G. Lai

**Affiliations:** 1 Institute of Health Informatics, University College London, London, UK; 2 Department of Health and Community Sciences, Faculty of Health and Life Sciences, University of Exeter, Exeter, UK; 3 UCL Cancer Institute, University College London, London, UK; 4 University College London Hospitals NHS Trust, London, UK

**Keywords:** Cancer, Diseases, Health care

## Abstract

A comprehensive evaluation of the total burden of morbidity endured by cancer survivors remains unavailable. This study quantified the burden of 144 health conditions and critical care admissions across 26 adult cancers and treatment modalities in 243,767 adults. By age 60, top conditions ranked by fold difference (cumulative burden in survivors divided by cumulative burden in controls) were haematology, immunology/infection and pulmonary conditions. Patients who had all three forms of treatment (chemotherapy, radiotherapy and surgery) experienced a high cumulative burden of late morbidities compared with patients who received radiotherapy alone. The top five cancers with the highest cumulative burden of critical care admissions by age 60 were bone (12.4 events per 100 individuals [CI: 11.6-13.1]), brain (9.0 [7.5-10.5]), spinal cord and nervous system (7.2 [6.7-7.8]), testis (6.7 [4.9-8.4]) and Hodgkin lymphoma (4.4 [3.6-5.1]). Conditions that were associated with high excess years-of-life-lost were haematological conditions (9.6 years), pulmonary conditions (8.6 years) and immunological conditions or infections (7.8 years). As the population of cancer survivors continues to grow, our results indicate that it is important to tackle long-term health consequences through enacting data-driven policies.

## Abstract (web summary)

Here the authors evaluate the burden of 144 health conditions in adult cancer survivors, and show that the magnitude of late morbidities experienced by survivors varies according to the type of cancer and treatment, highlighting opportunities for optimising patient care

## Introduction

Cancer survivorship is a unique period that differs across individuals and populations. The National Coalition for Cancer Survivorship employs the term cancer survivor to indicate a person who has ever been diagnosed with cancer, regardless of where they are in the course of their disease. According to a systematic review, the most widely used definition considers cancer survivorship as a phase that begins at the point of diagnosis^1^. The survivorship phase continues to pose a challenge against implementing effective policies to ensure that all aspects of an individual’s wellbeing are taken care of as the focus now shifts from cancer treatment to managing long-term side effects. The European Cancer Patient Coalition recommends^2^ that more research on survivorship is needed to return data on the late side-effects of treatment and the risks of developing subsequent cancer to help inform guidelines on long-term follow-up care. Yet, there has been a lack of systematic approaches to generating data insights on the late morbidities, which is in part due to limitations in establishing a reference phenotype catalogue of a wide range of conditions in a manner that could drive scalable data analytics across conditions and clinical specialties. With the availability of population, disease agnostic, health records of high clinical granularity, it is now possible to develop a phenome-wide systematic map of late morbidities for patient benefit.

A validated electronic health record phenotype catalogue is readily available^3^ but has not been applied on a population-level dataset to generate estimates on the overall burden of late morbidities and their effects on prognosis across all major cancer types and cancer treatments. Such real-world data-driven insights are likely to have the largest impact on survivorship care and may inform clinical practice guidelines. Someone who survived cancer might expect healthcare systems to learn from the vast amount of previous data on other ‘*patients like them*’^4^ and inform health strategies that are most likely to deliver benefits to patients. The National Cancer Registration and Analysis Service publish a large collection of reports on cancer and its treatment^5^, however, these reports are not patient-centred as they do not focus on understanding patients beyond cancer. Large-scale analyses on non-cancer comorbidities are limited. Many of these conditions are treatable, however, they still contribute significantly to morbidity and premature mortality. For example, over 10% of patients with cancer do not die from their cancer, but heart disease^6^. In light of these consequences, a proactive approach for preventing, identifying and treating diseases is to start at the point of a cancer diagnosis before cancer treatment is administered.

Here, we address the unmet need for risk and prognostic information of late morbidities among cancer survivors through a phenome-wide lens using real-world data from routinely collected electronic health records. Specific objectives are: (1) to estimate the total burden of 144 health conditions, spanning 12 organ systems in cancer survivors and controls and by socioeconomic deprivation statuses, (2) to estimate the cumulative burden of health conditions across 26 cancer types, seven treatment exposures and 10 chemotherapy drug classes, (3) to examine the risk of developing late morbidities using regression analysis, (4) to estimate the burden of critical care admission by cancer type and treatment exposure, (5) to estimate excess years of life lost attributable to a particular health condition and (6) to develop effective visualisations to present large amounts of actionable information. This comprehensive study aims to fill an important gap in cancer survivorship care related to identifying patients who might benefit from screening and treatment of late morbidities. There is evidence that over-screening in cancer survivors might lead to more harm due to overdiagnosis^7^, thus, our results may inform survivorship screening guidelines to limit screening to patients whose prognosis could benefit from early detection and to promote adherence to screening in patients who need them the most.

## Results

Between 1998 to 2020, 243,767 adults with an incident site-specific cancer diagnosis and who survived for at least 1 year were identified (Supplementary Data 1, Supplementary Fig. 1). Cancer survivors were matched to 506,892 controls with no history of cancer from the general population. The mean ages for cancer survivors (66.7 years) and matched controls (66.6 years) were comparable. A total of 48.3% and 49.1% of cancer survivors and controls were men, respectively. The median follow-up time for cancer survivors was 5.1 years (interquartile range [IQR] 7.6 years). For controls, median follow-up was 8.5 years (IQR 9.0 years). 26.2% and 25.4% of cancer survivors and controls, respectively, were from the highest socioeconomic group according to the index of multiple deprivation (IMD). 14.4% and 15.2% of survivors and controls, respectively, were from the lowest socioeconomic group.

Cancer survivors were grouped according to 26 site-specific diagnostic categories: bladder (9652 individuals), bone (343), brain (1614), breast (57,365), cervix (2547), colon and rectum (35,054), gallbladder and biliary tract (744), Hodgkin lymphoma (1286), kidney and renal pelvis (6374), leukaemia (5747), liver and intrahepatic bile duct (1063), lung and bronchus (13,993), melanoma (12,278), multiple myeloma (3765), non-Hodgkin lymphoma (10,163), oesophagus (3998), oropharynx (5491), ovary (5816), pancreas (1696), prostate (47,614), small intestine (710), spinal cord and nervous system (92), stomach (3764), testis (1921), thyroid (1937), and uterus (8740). In terms of treatment, 57.0% of survivors had surgery, 26.5% had chemotherapy and 30.1% had radiotherapy. 33.0% of survivors only had surgery without chemotherapy or radiotherapy, 8.1% only had chemotherapy and 8.5% only had radiotherapy. 5.9% had all three forms of treatment (chemotherapy, radiotherapy and surgery).

### Cumulative burden of late morbidities in cancer survivors and controls by deprivation status

**Fig. 1:**
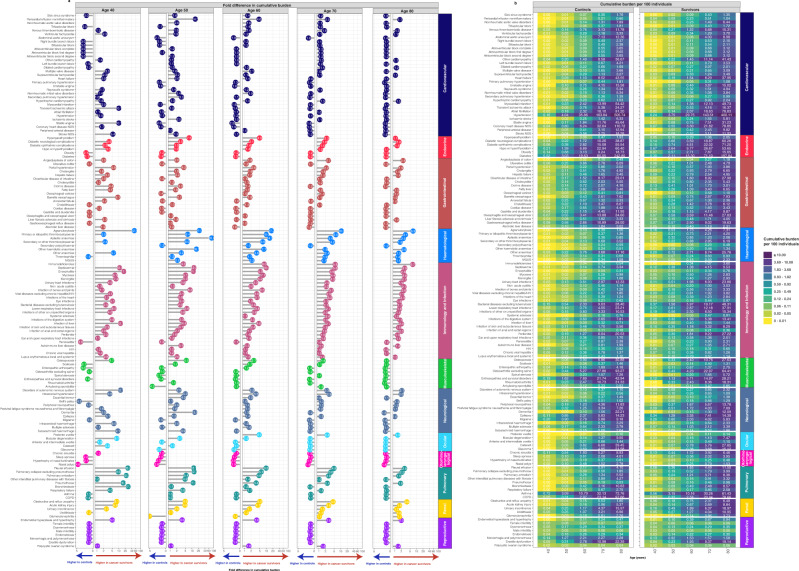
Cumulative burden of health conditions for cancer survivors and controls from the highest socioeconomic group based on the Index of Multiple Deprivation (IMD). Health conditions were rank-ordered according to the fold difference at age 60 in individuals from the lowest socioeconomic group (see corresponding results for the lowest socioeconomic group in Supplementary Fig. 1). **a** Lollipop graphs depict the fold difference of cumulative burden in survivors versus controls for different age groups. The fold difference is annotated in each lollipop. Conditions that have a higher cumulative burden in survivors are shown on the right side within each age-specific plot, while conditions with a higher cumulative burden in controls are shown on the left. Where there are no events in either survivors or controls, the fold difference is not calculated. **b** Heatmaps depict cumulative burden counts per 100 individuals for survivors and controls. Each tile in the heatmap corresponds to cumulative burden count per 100 persons for each condition-specific outcome at different age groups. For example, a cumulative burden of 11.78 for venous thromboembolic disease in controls at age 80 corresponds to 11.78 events per 100 individuals. Cumulative burden values were separated into 10-quantiles (10 groups) resulting in quantile colour representation of the heatmaps. Source data are provided as a Source Data file.

We observed that for many health conditions, the cumulative burdens of morbidities were higher in survivors compared with controls, with some differences when comparing across age groups (Fig. 1, Supplementary Data 2, Supplementary Data 3). Similar trends were observed among individuals from the highest socioeconomic status (Fig. 1) and lowest socioeconomic status (Supplementary Fig. 2). Among individuals from the highest socioeconomic group, by age 50 years, pericardial effusion was 14 times higher in cancer survivors (0.090 events per 100 individuals [CI: 0.081–0.099]) compared with controls (0.006 [0.005–0.008]) (Fig. 1, Supplementary Data 3). Similarly, pericardial effusion was seven times higher in cancer survivors (0.201 [0.153–0.249]) from the lowest socioeconomic group compared with controls (0.026; [0.025–0.028]) (Supplementary Fig. 2, Supplementary Data 3).

Among individuals from the highest socioeconomic group, by age 60, top conditions ranked by fold difference (cumulative burden in survivors divided by cumulative burden in controls) within each organ system were: cardiovascular (pericardial effusion: 4.6-fold higher in cancer survivors compared with controls), endocrine (hyperparathyroidism: 4.9-fold), gastrointestinal (portal hypertension: 2.6-fold), haematological (agranulocytosis: 17.2-fold), immunology and infection (septicaemia: 10.0-fold), musculoskeletal (osteoporosis: 2.7-fold), neurological (intracranial hypertension: 3.3-fold), ocular (posterior uveitis: 4.7-fold), pulmonary (pleural effusion: 9.8-fold), renal (obstructive and reflux uropathy: 5.0-fold) and reproductive (endometrial hyperplasia and hypertrophy: 3.6-fold) (Fig. 1a). Figure 1b depicts the cumulative burden per 100 cancer survivors or controls within the highest socioeconomic group at ages 40, 50, 60, 70, and 80.

By contrast, among individuals from the lowest socioeconomic group, by age 60, conditions with the highest fold-difference in cumulative burden were: cardiovascular (sick sinus syndrome: 3.6-fold higher in cancer survivors than in controls), endocrine (hyperparathyroidism: 1.9-fold), gastrointestinal (angiodysplasia of colon: 2.9-fold), haematological (agranulocytosis: 15.5-fold), immunology and infection (immunodeficiencies: 25.5-fold), musculoskeletal (osteoporosis: 2.8-fold), neurological (disorders of autonomic nervous system: 2.9-fold), ocular (posterior uveitis: 15.8-fold), pulmonary (pleural effusion: 6.4-fold), renal (obstructive and reflux uropathy: 7.4-fold), and reproductive (endometrial hyperplasia and hypertrophy: 2.9-fold) (Supplementary Fig. 2a). Supplementary Fig. 2b depicts the cumulative burden per 100 cancer survivors or controls within the lowest socioeconomic group at ages 40, 50, 60, 70, and 80. Complete data for cumulative burden along with confidence intervals are provided in Supplementary Data 3.

### Variations in cumulative burden of health conditions by age across 26 site-specific cancers

We estimated the cumulative burden of 144 health conditions (categorised into 12 organ system groups) across 26 cancer types. When considering endocrine-related morbidities, patients with thyroid cancer, by age 60, had 275.8 hypo- or hyperthyroidism events per 100 individuals (CI: 206.8–344.8) (Supplementary Data 4). By contrast, patients with prostate cancer only had 0.52 (0.27–0.77) hypo- or hyperthyroidism events. For immunology and infection conditions, patients with Hodgkin lymphoma had the highest disease burden while for neurological conditions, patients with brain cancer had the highest burden. Patients with Hodgkin lymphoma had 38.1 bacterial infection events per 100 individuals (CI: 27.22–48.94) and 24.9 events (22.3–27.5) related to lower respiratory tract infection (Supplementary Data 4). By comparison, the cumulative burden of bacterial infections in patients with lung cancer was 3.2 (3.1–3.3) and the cumulative burden of lower respiratory tract infection in patients with bladder cancer was 1.6 (1.3–1.8). For neurological conditions, the cumulative burden for epilepsy and intracranial hypertension in patients with brain cancer by age 60 was 234.7 events per 100 individuals (CI: 216.2–253.2) and 7.5 (7.2–7.8), respectively. By contrast, the cumulative burden for epilepsy in patients with bladder cancer was 0.3 (0.2–0.3), and the burden for intracranial hypertension at the same age in patients with oesophageal cancer was 0.03 (0.00–0.05).

When exploring specific cancer types, in patients with liver cancer aged 60 for example, the top five conditions with the highest burden were diabetes (33.7 events per 100 individuals [29.3–38.2]), hypertension (33.2 [30.6–35.9]), chronic viral hepatitis (21.2 [17.4–25.1]), liver fibrosis and cirrhosis (20.9 [15.7–26.2]) and infection of the liver (19.4 [14.5–24.3]) (Supplementary Data 4). In patients with leukaemia at age 60, the top five conditions were diabetes (42.1 events per 100 individuals [37.6–46.7]), hypertension (38.6 [37.6–39.6]), asthma (21.0 [15.7–26.2]), lower respiratory tract infections (18.0 [18.0–18.0]) and bacterial diseases (17.7 [15.3–20.1]). Immunological conditions or infections and haematological conditions were also prominent in patients with leukaemia, for example agranulocytosis (11.3 [11.2–11.3]), viral diseases (8.1 [7.8–8.3]), secondary thrombocytopaenia (7.7 [7.0–8.4]), septicaemia (7.5 [6.9–8.2]) and primary or idiopathic thrombocytopaenia (7.1 [4.4–9.8]) (Supplementary Data 4).

### Fold difference of cumulative burden of health conditions across 26 site-specific cancers

**Fig. 2:**
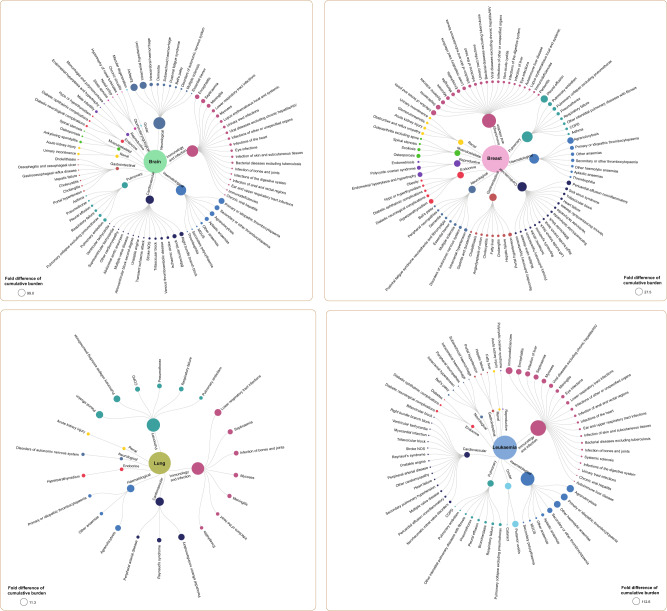
Cumulative burden of health conditions among cancer survivors at age 60 across 26 cancer types. Circular dendrograms depict the fold difference of cumulative burden in survivors versus controls where conditions with a fold difference of ≥2 are shown. Fold difference for health conditions in patients with leukaemia, brain, breast and lung cancers are shown. The area of the nodes is proportional to the fold difference of each condition, and the conditions are ranked from the highest to lowest fold difference. For example, for brain cancer, neurological conditions exhibit the highest fold difference in cumulative burden in cancer survivors versus controls, and within this group, epilepsy has the highest fold difference. Dendrograms for all other cancer types are shown in the supplementary figures. Source data are provided as a Source Data file.

By age 60 years, epilepsy was 99.02 (CI: 89.17–109.78) times higher in brain cancer survivors compared with controls (Fig. 2, Supplementary Data 5). Intracranial hypertension and intracerebral haemorrhage were 93.50 (82.52–107.44) times and 22.25 (19.08–25.59) times higher in brain cancer survivors, respectively. Among survivors of breast cancer, bone infection, septicaemia and systemic sclerosis were 13.78 (13.11–14.44) times, 11.36 (10.12–12.71) times and 8.40 (2.18–15.32) times higher, respectively, compared with controls. Whereas among survivors of lung cancer the fold increases of pulmonary-related conditions were as follows: pleural effusion (11.35 [9.46–13.97]), pulmonary collapse (9.78 [8.71–11.09]), chronic obstructive pulmonary disease (5.27 [5.13–5.42]), respiratory failure (4.70 [2.81–6.98]) and pulmonary embolism (2.69 [1.94–3.58]). Survivors of leukaemia experienced an increase in several haematological conditions. The fold increases were as follows: agranulocytosis (112.60 [102.35–125.12]), primary or idiopathic thrombocytopaenia (101.00 [55.55–191.90]), haemolytic anaemias (96.33 [40.85–305.95]) and secondary thrombocytopaenia (59.08 [44.42–86.09]). Fold differences for other cancers are shown in Supplementary Data 5 and Supplementary Figs. 3 to 23.

### Variations in cumulative burden of health conditions by follow-up time across 26 site-specific cancers

**Fig. 3:**
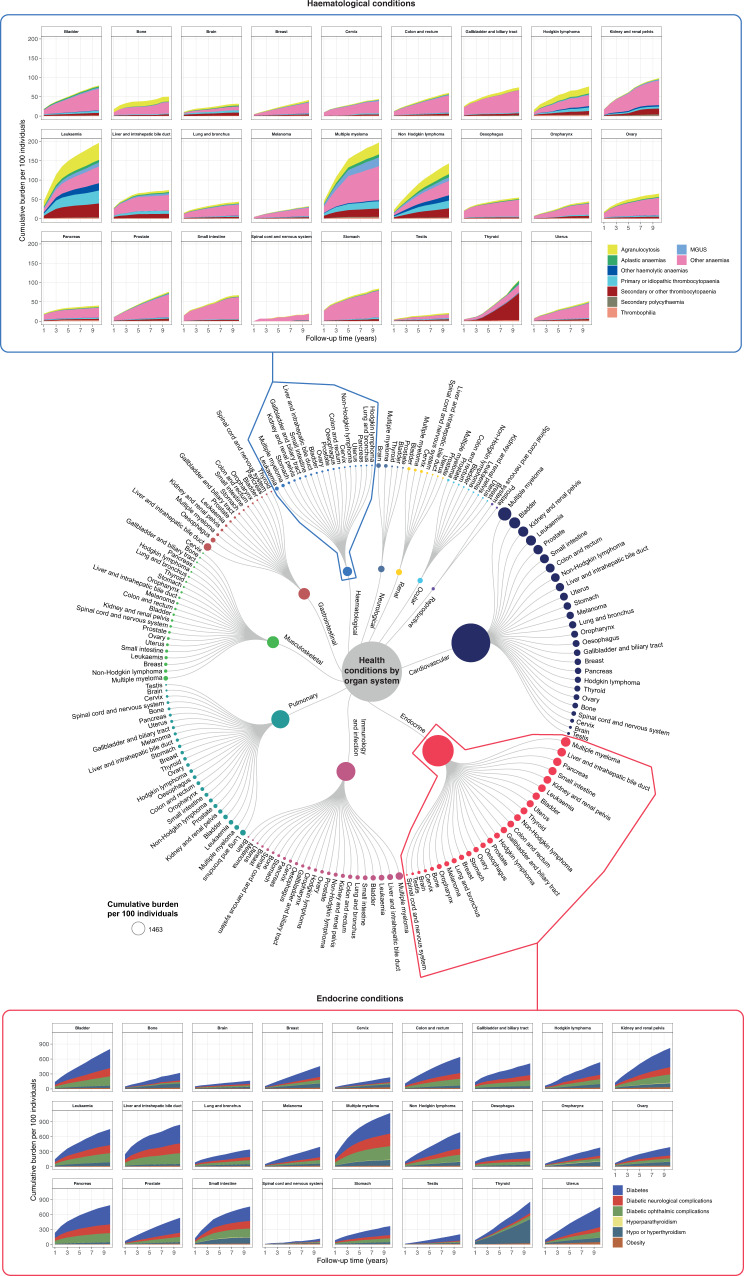
Cumulative burden of health conditions among cancer survivors across organ systems. Cumulative burden per 100 individuals according to follow-up time is shown. Circular dendrogram in the centre depicts the cumulative burden of health conditions at the organ system level at 5 years of follow-up. Within each organ system, cancer types are ranked from the highest to lowest burden. For example, when considering pulmonary conditions, patients who had lung cancer have the highest disease burden. The circular dendrogram only displays conditions with at least 20 events per 100 individuals. Area charts above and below the circular dendrogram display the cumulative burden of individual haematological conditions and endocrine conditions, respectively. Cumulative burden of individual conditions is shown according to follow-up time. Source data are provided as a Source Data file. Area graphs for other organ systems are provided as supplementary figures.

We estimated the cumulative burden of health conditions according to follow-up time. At five years of follow-up, among survivors of renal cancer, conditions with the highest burden were as follows: hypertension (456.74 events per 100 individuals [438.03–475.46]), diabetes (224.62 [211.48–237.76]), chronic obstructive pulmonary disease (106.26 [104.1–108.43]), diabetic ophthalmic complications (103.74 [88.8–118.68]) and coronary heart disease (102.67 [93.24–112.11]) (Supplementary Data 6). Whereas, among survivors of liver cancer, the cumulative burden of morbidities at five years of follow-up were as follows: hepatic failure (91.56 [74.63–108.48]), infection of the liver (52.76 [48.3–57.23]), myocardial infarction (52.64 [51.81–53.48]), atrial fibrillation (51.20 [48.98–53.42]) and portal hypertension (40.16 [38.73–41.6]). When comparing across haematological conditions, survivors of leukaemia, multiple myeloma and non-Hodgkin lymphoma had the highest disease burden (Fig. 3). For example, survivors of leukaemia exhibited a high burden of agranulocytosis (34.59 [32.11–37.06]), anaemia (29.59 [27.77–31.41]) and secondary thrombocytopaenia (28.93 [21.86–35.99]) at five years of follow-up (Supplementary Data 6). When considering renal conditions, survivors of bladder cancer had a high burden of urinary incontinence (35.16 [33.23–37.10]), obstructive and reflux uropathy (21.69 [21.10–22.29]), acute kidney injury (16.28 [15.54–17.03]) and glomerulonephritis (4.84 [3.25–6.43]). Complete data for cumulative burden according to follow-up time are provided in Supplementary Data 6 and are graphically represented in Supplementary Figs. 24 to 33.

### Variations in cumulative burden of health conditions across seven cancer treatment exposures

**Fig. 4:**
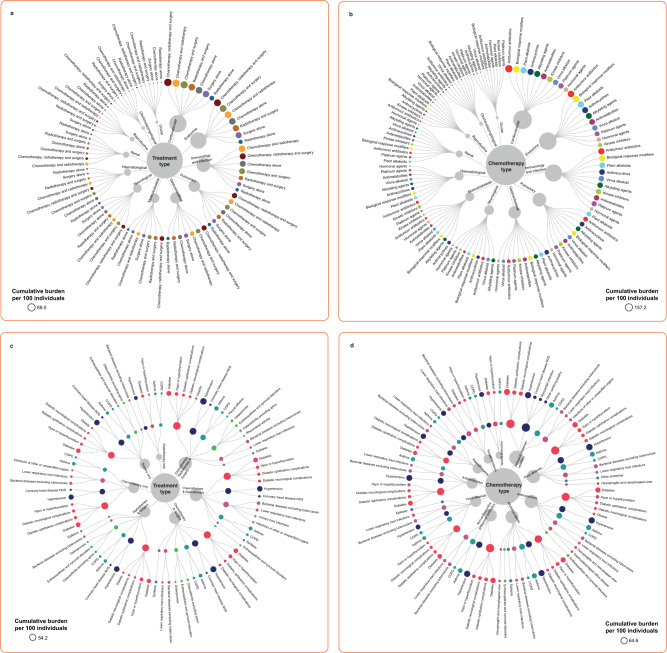
Cumulative burden of health conditions among cancer survivors at age 60 by treatment type and chemotherapy agents. Cumulative burden per 100 individuals is shown. Dendrograms depict the cumulative burden of conditions where the top hierarchy is organ system followed by **a** treatment type or **b** chemotherapy type. This allows the visualisation of the burden of health conditions for each treatment type or chemotherapy type ranked from the highest to lowest. The area of the nodes is proportional to the burden of health conditions within organ systems. Dendrograms depict the disease burden at the level of individual conditions by **c** treatment type or **d** chemotherapy type, and the conditions are ranked from highest to lowest burden. The area of the nodes is proportional to the burden of each condition. The treatment type dendrogram in **c** displays conditions with at least 5 events per 100 individuals. The chemotherapy agent dendrogram in **d** displays conditions with at least 10 events per 100 individuals. Source data are provided as a Source Data file.

Having received all three forms of treatment (chemotherapy, radiotherapy, and surgery) was associated with a high cumulative burden of health conditions. By age 60, conditions with high cumulative burdens were hypertension (54.2 events per 100 individuals [51.2–57.1]), diabetes (31.5 [27.5–35.5]), hypo- or hyperthyroidism (16.8 [16.4–17.1]), bacterial diseases (14.7 [14.4–15.0]), osteoporosis (10.7 [9.5–11.9]), lower respiratory tract infections (7.9 [7.3–8.5]) and anaemia (7.5 [7.1–7.9]) (Fig. 4, Supplementary Data 7). By contrast, patients who received only radiotherapy had an overall lower burden of health conditions: hypertension (18.9 [18.0–19.7]), diabetes (11.8 [10.7–12.9]), hypo- or hyperthyroidism (5.6 [5.2–6.0]), epilepsy (4.4 [3.8–5.1]), oesophagitis and oesophageal ulcer (3.6 [3.5–3.7]), lower respiratory tract infection (3.2 [3.0–3.4]) and heart failure (2.4 [2.0–2.8]).

Patients who received chemotherapy (alone, or in combination with radiotherapy and/or surgery) exhibited a higher cumulative burden of myocardial infarction compared with patients who received only radiotherapy and/or surgery. The cumulative burdens of myocardial infarction in patients who had chemotherapy were chemotherapy and radiotherapy (4.7 [3.5–6.0]), chemotherapy, radiotherapy and surgery (3.8 [2.7–5.0]) and chemotherapy and surgery (3.3 [2.8–3.7]) (Fig. 4, Supplementary Data 7). Cumulative burden of myocardial infarction in patients who only had chemotherapy (4.2 [3.8–4.6]) was nearly twice that of patients who had radiotherapy and surgery but not chemotherapy (2.3 [2.0–2.6]). Chemotherapy was also associated with a higher burden of heart failure events; chemotherapy and radiotherapy (3.3 [3.2–3.3]), chemotherapy, radiotherapy and surgery (3.1 [2.8–3.5]) and chemotherapy and surgery (2.9 [2.5–3.3]). By contrast, cumulative burden of heart failure was two times lower in patients who only received surgery (1.5 [1.4–1.6]) than in those who received chemoradiation.

### Variations in cumulative burden of health conditions across 10 chemotherapy drug classes

We observed that patients who received biological response modifiers or immunotherapies had the highest burden of health conditions overall, while those who received hormonal agents had the lowest disease burden. By age 60, cumulative burden of health conditions in patients who had biological response modifiers or immunotherapies versus hormonal agents were as follow: hypertension (61.2 events per 100 individuals [CI: 60.2–62.3] vs. 36.4 [35.7–37.1]), diabetes (45.6 [39.3–52.0] vs. 21.5 [20.7–22.2]), bacterial diseases (21.8 [20.4–23.2] vs. 8.9 [8.7–9.0]), hypo- or hyperthyroidism (19.9 [13.5–26.2] vs. 12.2 [11.6–12.8]), lower respiratory tract infection (17.3 [15.0–19.5] vs. 5.3 [5.0–5.6]), peripheral neuropathies (8.4 [7.6–9.1] vs. 3.5 [3.0–4.0]), agranulocytosis (8.3 [7.8–8.7] vs. 2.7 [2.6–2.8]), septicaemia (8.2 [7.7–8.6] vs. 2.6 [2.6–2.6]) and heart failure (6.4 [5.1–7.7] vs. 1.8 [1.5–2.1]) (Fig. 4, Supplementary Data 8).

### Cumulative burden of critical care admissions by primary cancer diagnosis, treatment exposure and chemotherapy type

When comparing across primary cancer diagnostic groups, the top five cancers with the highest cumulative burden of critical care admissions by age 60 were bone (12.4 events per 100 individuals [CI: 11.6–13.1]), brain (9.0 [7.5–10.5]), spinal cord and nervous system (7.2 [6.7–7.8]), testis (6.7 [4.9–8.4]) and Hodgkin lymphoma (4.4 [3.6–5.1]) (Supplementary Fig. 34a, Supplementary Data 9). By contrast, cumulative burdens of critical care admissions were low in prostate (0.1 [0.1–0.1]), lung (0.5 [0.4–0.6]) and stomach cancers (0.5 [0.3–0.7]).

Cumulative burden of critical care admissions was consistently higher in patients who received chemotherapy alone or in combination with other treatments. By age 60, the cumulative burdens were chemotherapy, radiotherapy and surgery (2.4 events per 100 individuals [2.3–2.5]), chemotherapy and radiotherapy (2.3 [2.1–2.5]) and chemotherapy and surgery (1.8 [1.8–1.8]). By contrast, the burden of critical care admissions was two-fold lower in patients who had surgery only (0.9 [0.9–1.0]) or radiotherapy only (0.7 [0.7–0.7]) compared with those who had chemotherapy only (1.7 [1.6–1.7]) (Supplementary Fig. 34B, Supplementary Data 10).

Among patients who received chemotherapy, by age 60, those who were treated with plant alkaloids excluding vinca alkaloids (3.4 [3.2–3.6]) or platinum agents (3.3 [3.2–3.5]) had the highest burden of critical care admissions at age 60, while those who received hormonal agents (1.2 [1.0–1.3]) had the lowest burden (Supplementary Fig. 34C, Supplementary Data 11).

### Multivariable logistic regression analysis for the association between treatment exposure and diagnosis of health conditions

**Fig. 5:**
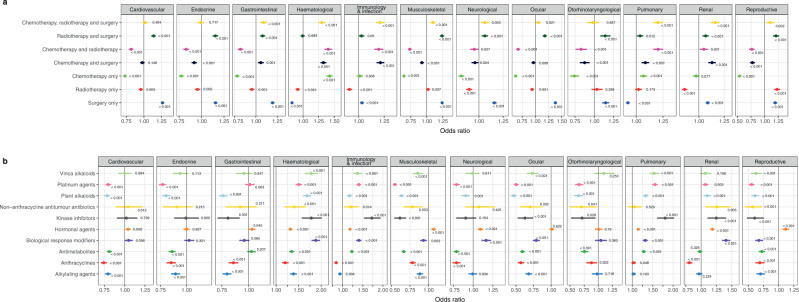
Multivariable logistic regression analysis of health condition outcomes among cancer survivors by different exposures. **a** Treatment type. **b** Chemotherapy agent. Forest plots show data presented as odds ratios and error bars represent 95% confidence intervals. *P* values are indicated on the plot. The Wald test is employed. Source data are provided as a Source Data file. The number of patients within each treatment category were as follows: chemotherapy only (19,691), surgery only (80,491), radiotherapy only (20,662), chemotherapy and radiotherapy (12,399), chemotherapy and surgery (18,125), radiotherapy and surgery (25,909) and chemotherapy, radiotherapy and surgery (14,349). The number of patients within each chemotherapy category were as follows: alkylating agents (7795), anthracyclines (6858), antimetabolites (12,628), biological response modifiers (7852), hormonal agents (22,304), kinase inhibitors (1187), non-anthracycline antitumour antibiotics (727), plant alkaloids excluding vinca alkaloids (7707), platinum agents (9570), and vinca alkaloids (2821).

Exposure to all three treatments (chemotherapy, radiotherapy and surgery) was associated with a greater likelihood of developing gastrointestinal (1.09 [1.05–1.13]), haematological (odds ratio [OR]: 1.30 [CI: 1.25-1.35]), musculoskeletal (1.10 [1.06–1.14]), neurological (1.06 [1.02–1.11]), ocular (1.05 [1.01–1.10]), pulmonary (1.20 [1.16-1.25]), renal (1.16 [1.12–1.21]), reproductive (1.10 [1.03–1.17]) and immunological conditions or infections (1.21 [1.17–1.25]) (Fig. 5a, Supplementary Data 12). Having received kinase inhibitors was associated with a greater likelihood of developing pulmonary (OR: 1.81 [CI: 1.61–2.03]), haematological (1.78 [1.57–2.01]), renal (1.25 [1.10–1.41]) and immunological conditions or infections (1.72 [1.53–1.94]) (Fig. 5b, Supplementary Data 13). Exposure to hormonal agents was associated with a higher risk of having reproductive issues (1.12 [1.06–1.17]). Patients who were treated with alkylating agents (0.93 [0.89–0.98]) or anthracyclines (0.84 [0.80–0.88]) had a lower risk of developing immunological conditions or infections. By contrast, those treated with kinase inhibitors (1.72 [1.53–1.94]), biological response modifiers or immunotherapies (1.40 [1.34–1.47]), platinum agents (1.40 [1.34–1.46]) and vinca alkaloids (1.37 [1.27–1.48]) experienced higher risks.

### Excess years of life lost attributable to late morbidities among cancer survivors

**Fig. 6:**
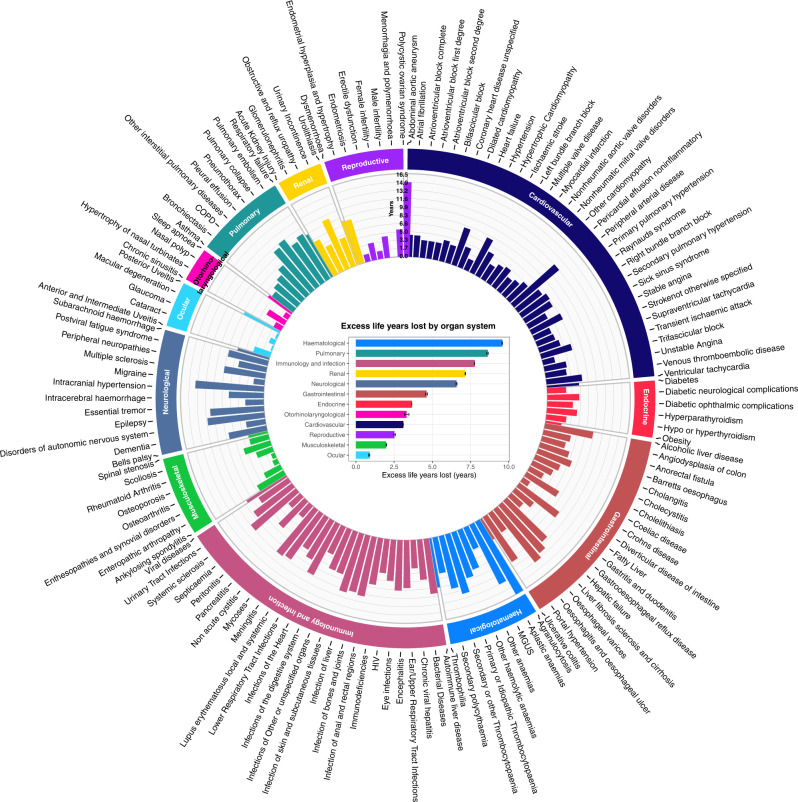
Excess years of life lost (YLL) attributable to late morbidities in cancer survivors. Circos plot depicts the difference in YLL between survivors who developed a health condition compared with survivors who did not develop a health condition. Bar chart depicts excess YLL for conditions grouped by organ system. Data are presented as excess years of life lost and error bars represent 95% confidence intervals. The number of cancer survivors was 243,767. Source data are provided as a Source Data file.

Excess years of life lost (YLL) was defined as the average number of years that cancer survivors with late morbidity lose in excess of that found in patients without that condition. Excess YLLs were displayed as a circos plot to visualise differences across 144 conditions (Fig. 6, Supplementary Data 14). Conditions with high excess YLL were agranulocytosis (16.30 years [CI: 16.25–16.34]), intracranial hypertension (13.57 [13.34–13.81]), oesophageal varices (13.32 [13.28–13.35]), aplastic anaemias (13.16 [12.84–13.48]), liver fibrosis sclerosis and cirrhosis (12.46 [12.32–12.60]), primary or idiopathic thrombocytopaenia (12.36 [12.28–12.45]), infection of liver (12.12 [12.12–12.13]) and pericardial effusion (11.66 [11.38–11.94]) (Fig. 6, Supplementary Data 14).

We also explored premature mortality by organ system and observed that haematological conditions (9.58 [9.55–9.61]), pulmonary conditions (8.63 [8.56–8.69]) and immunological conditions or infections (7.78 [7.74–7.82]) were associated with high excess YLL (Fig. 6, Supplementary Data 15). By contrast, patients who developed ocular (0.91 years [CI: 0.82–0.99]) or musculoskeletal (1.98 [1.97–1.98]) conditions only experienced modest excess YLL.

## Discussion

The advancement of cancer therapy over the last few decades has resulted in improved overall survival. However, therapeutic improvements have not eliminated late adverse events and cancer- or treatment-associated sequelae continue to threaten survivorship. This study provides new insights in several ways. To our knowledge, there has not yet been any study performing comprehensive assessments of health status and the impact of treatment-related morbidity within a single cohort across 26 adult cancers at a scale that leverages multiple sources of linked health records from a public healthcare system. We employed different forms of visualisations to effectively present large amounts of data in a manner that allows the identification of actionable patterns. Employing a framework utilising open-access electronic health record phenotypes, we not only systematically described the phenome-wide cumulative burden of 144 conditions, but also and importantly, the magnitude of variation across cancer diagnostic groups, treatment modalities, chemotherapy drug classes and socioeconomic statuses. The overall burden of late adverse events in cancer survivors far surpasses, by several orders of magnitude, events observed in matched controls. Detailed understanding of late morbidities among cancer survivors may facilitate the earlier diagnoses of these conditions, many of which are treatable.

We observed large increases in the burden of haematological and immunological conditions or infections among cancer survivors across all ages compared with controls. When looking across 26 cancer diagnostic groups, cardiovascular (hypertension, coronary heart disease, myocardial infarction and heart failure) and endocrine issues (diabetes, hypo- or hyperthyroidism) were also common. A US-based study conducted via the Surveillance, Epidemiology, and End Results program found that 11% of cancer patients do not die from cancer but cardiovascular disease^6^. Hypertension and diabetes may contribute to elevated risks of atherosclerosis leading to myocardial infarction or stroke. We found that treatment with biological response modifiers or immunotherapies, plant alkaloids, anthracyclines or alkylating agents were associated with an increased burden of hypertension. Poorly controlled hypertension could lead to further damage via chemotherapy-induced heart failure^8^. A randomised trial demonstrated the benefit of intensive lifestyle intervention using diet, exercise and sodium restriction for reducing blood pressure in patients with resistant hypertension^9^, suggesting that structured intervention in a cancer rehabilitation setting could be effective in lowering blood pressure without the risks of side effects from antihypertensive medications.

The presentation of late morbidities differed according to the initial cancer diagnosis. Patients with brain cancer experienced a high burden of epilepsy and intracranial hypertension. Patients with haematological malignancies such as leukaemia experienced a high burden of agranulocytosis, thrombocytopaenia and septicaemia. Whereas, in patients with liver cancer, hypertension, liver infection and liver fibrosis and cirrhosis were prominent. In terms of treatment effects, exposure to chemoradiation can lead to musculoskeletal issues and rheumatic presentations. We found that patients who were exposed to chemoradiation experienced a high burden of osteoporosis, osteoarthritis and rheumatoid arthritis. Furthermore, treatment with hormonal chemotherapeutic agents could worsen bone loss. Over 42% of patients with prostate cancer who received androgen-deprivation therapy experienced osteoporosis after two years^10^. Treatment of breast cancer with aromatase inhibitors with ovarian suppression was effective in reducing recurrence, however, osteoporosis was found in 13% of patients^11^. Similarly, treatment with another aromatase inhibitor, letrozole, was associated with an increased incidence of bone fracture in 9% of patients^12^. Other forms of chemotherapy may cause bone damage through indirect systemic effects. Adjuvant cyclophosphamide, methotrexate and fluorouracil therapy contributed to ‘chemical castration’ or early menopause and rapid bone loss in premenopausal breast cancer patients^13^. By the second year, 9.5% of patients experienced bone loss at the lumbar spine and 4.6% at the femoral neck^13^. Radiation, on top of chemotherapy, could worsen damage to bone tissue. Radiation exposure damages bone quality by affecting the trabecular architecture, which parallels with adipocyte infiltration of the bone marrow to alter the composition of the microenvironment affecting overall bone quality^14^. Mitigating cancer-induced bone loss can be achieved by reducing osteoclast activity, which are specialised cells that absorb and removes bones. Treatment with zoledronic acid (a bisphosphonate) could also reduce the risk of bone loss^15^.

Our results may inform approaches for optimising coordinated care starting from the point of cancer diagnosis. Many cancer survivors feel that their lives are radically altered by cancer. Approximately 1.8 million individuals in England currently have a cancer diagnosis and this figure is projected to rise to 3 million by 2030^16^. We and others have shown that cancer survivors continue to face significant health challenges that could have been managed or avoided during the survivorship period^17–19^. The UK’s National Cancer Survivorship Initiative (NCSI) emphasised the need for an evidence base about what long-term consequences survivors might face to address gaps in service provision^20^. Key actions proposed by NCSI include (1) supporting patients in making decisions about treatment options, (2) promoting cancer rehabilitation to prevent avoidable health consequences, (3) offering patients a tailored care plan, (4) equipping patients to self-manage their condition with lifestyle changes, and (5) assessment and monitoring of consequences of treatment during the active treatment phase and survivorship period^20^.

Follow-up for cancer patients typically occurs in a hospital outpatient setting, however, a systematic review demonstrated that there was no difference between primary and secondary care follow-up in terms of morbidity and patient wellbeing although the authors caution against poor data quality^21^. Thus, care pathways could usefully employ the coordinated care approach to reduce the burden of hospital outpatient activity. GPs are the primary point of contact for patients in the UK, and with adequate training, low-risk follow-up could be GP-led, although for this to happen there would need to be a programme of education and training, excellent two-way communication between primary and secondary care, personalised treatment and management plans, and incentivisation. For example, the National Institute for Health and Care Excellence (NICE) guideline for prostate cancer recommends follow-up in primary care and for the active surveillance of the prostate-specific antigen kinetics^22^. Nonetheless, coordinated care between healthcare providers may present additional challenges in terms of information exchange and care continuity. A qualitative content analysis of over 400 documents identified 70 correspondence-related items between primary and secondary care about patients with cancer^23^. Notably, these documents often included less relevant information where rarely did letters include information about treatment intent, treatment alternatives or how patients reacted to the information given. The authors concluded that letters were not tailored to the needs of the patient and were not written with the aim of mutual communication^23^.

The quality of life of many cancer survivors remains poor as toxicities from cancer therapies can cause new chronic conditions to emerge. For example, we observed a significant burden of cardiovascular disease among patients, suggesting that effective strategies to mitigate cardiovascular risk are needed. The American Heart Association (AHA) proposed a multimodal solution, which involves identifying patients who are at high risk for cardiac dysfunction and harnessing cardiac rehabilitation (i.e., prescriptive exercise, behavioural interventions and cardiac risk factor modification)^24^. Importantly, the AHA guideline highlighted strategies for identifying patients who may benefit from multimodal cardiac rehabilitation. There has been limited consensus on which cancer therapies might predispose patients to a higher risk of cardiac dysfunction. However, the American Society of Clinical Oncology guideline^25^ states that patients who were treated with high-dose anthracycline or radiotherapy when the heart is in the treatment field, or treatment with low-dose anthracycline plus the presence of two or more cardiovascular risk factors or have compromised cardiac function should be considered at increased risk. We also observed a high burden of urinary incontinence in patients with cervical cancer. Chemoradiation poses additional risks of weakening the pelvic floor muscle causing issues such as urinary incontinence^26^. Anticholinergics can be prescribed to treat bladder control issues which may improve the psychological wellbeing of survivors and overall quality of life; however, care must be taken to identify potential contraindications to anticholinergics. Collectively, these findings underscore the importance of cross-specialty, multidisciplinary involvement as a standard of care during survivorship.

In terms of strengths, this study uses population-based health records providing granular detail, statistical power and longevity of follow-up. To our knowledge, it is the largest study of late morbidities among cancer survivors that harnesses health information across multiple settings (primary care, secondary care, cancer registry, and the death registry), allowing the accurate ascertainment of conditions managed in both GPs and hospitals. Second, we analysed the cumulative burden of 144 health conditions across 26 cancer types, seven treatment exposures and 10 chemotherapy drug classes to reveal heterogeneity in disease trends that might be previously underappreciated in smaller-scale studies using one-disease-at-a-time approaches. We provide cumulative burden estimates at the single-disease level instead of returning estimates on disease composites; the latter approach may mask important variations. Third, we applied a consistent methodology for the estimation of cumulative burden that accounted for competing risks (an important consideration in a survivorship setting), allowing cross-comparison across cancer types and treatment exposures. Instead of quantifying cumulative incidence, a metric commonly used in many epidemiological studies that consider only the first event, we employed the cumulative burden approach to quantify the total burden of events^27^. Fourth, we returned information on risk (regression analysis) and prognosis (years of life lost). Excess years of life lost estimations were provided for 144 health conditions allowing healthcare practitioners and policymakers to identify individuals who are at risk for immediate intervention. Fifth, we explored the impact of socioeconomic deprivation on disease burden and premature mortality, which may feed into initiatives focused on underserved individuals for targeted surveillance and intervention. Sixth, the UK employs a publicly funded healthcare system meaning that results from this study could be translatable to other countries with similar economies and healthcare systems.

Several limitations are acknowledged. Cancer registration data were incomplete and insufficient for certain variables. There was a high degree of missingness in tumour grade and stage data which precluded analyses of their effects on late morbidities. Information on cancer disease state is not available. We have not investigated the effects of chemotherapy or radiotherapy dose on late morbidities. We have not assessed prescription data and did not evaluate the impact of treatments for late morbidities on survival outcomes. We have not considered the prescribing of medications that could minimise the side effects of cancer therapy (i.e., cardio-protective medications) as we do not have access to hospital-prescribed medications. We have not considered ethnicity due to insufficient data.

As the population of cancer survivors continues to grow, disease- and treatment-level estimates are becoming increasingly relevant for optimising health and care interventions. To address the gaps in survivorship management, the American Cancer Society (ACS) has outlined three priority areas: (1) provision of personalised information and referrals from cancer diagnosis onwards, (2) implementation of new interventions, and (3) implementation of routine needs assessments of survivors^28,29^. Given the high burden of chronic conditions among survivors and the impact of these conditions on mortality, future research could focus on the identification and management of these conditions through medications or the promotion of lifestyle changes (i.e., smoking cessation efforts). Polypharmacy through the use of prescription medications for treating late morbidities is common among cancer survivors^30^. Future research could focus on investigating the impact of polypharmacy on medication adherence, and the risks of adverse drug events and drug-drug or drug-disease interactions. To achieve a high-performing survivorship care system, we propose that intersectoral care involving primary and secondary care follow-up after hospital discharge is vital to minimise unnecessary interventions without compromising the quality of care.

## Methods

### Population health record data sources

This study employed electronic health record datasets from general practices, hospitals (Hospital Episode Statistics [HES]), the National Cancer Registration and Analysis Service (NCRAS) and death records from the Office for National Statistics (ONS) in England during the study period of 01/01/1998 to 31/10/2020. For secondary care HES linked records, we utilised data from in-patient admissions from the Admitted Patient Care dataset and critical care admissions from the Adult Critical Care dataset. Patient-level index of multiple deprivation (IMD), which is an area-based measure of socioeconomic deprivation was employed. Details on cancer treatment were obtained from the Radiotherapy (RTDS) and the Systemic Anti-Cancer Treatment (SACT) datasets.

This research complies with all relevant ethical regulations. Ethics approval was obtained from the Medicines and Healthcare products Regulatory Agency (19222). Data has been obtained according to the terms and conditions and data use complies with the terms and conditions of the Medicines Healthcare products Regulatory Agency Independent Scientific Advisory Committee. Patient informed consent was not required.

### Study design

We identified patients with 26 site-specific cancers. Incident cancer in patients aged 18 years or older was defined as the first diagnosis of cancer at a site of interest between 01-01-1998 and 31-10-2020. Cancer survivors were defined as patients who were alive one year after cancer diagnosis; patients who died within a year of cancer diagnosis were excluded. Controls were identified using propensity score matching. Controls were matched by year of birth, sex and Index of Multiple Deprivation. Matching was performed using the R matchit package and the nearest-neighbour matching algorithm was selected. Cancer survivors and controls were matched at a 1:2 ratio, with a caliper width of 0.2 of the standard deviation of the logit of the propensity score. Follow-up for cancer survivors started 1 year after cancer diagnosis (index date). Index dates of corresponding matched cancer survivors were used as the date of the start of follow-up for controls. All individuals were followed up until death, date of deregistration from the practice or date of administrative censoring (31-10-2020), whichever occurred first.

### Site-specific cancers, health conditions and treatment exposures

We analysed 26 cancer types: (1) bladder, (2) bone, (3) brain, (4) breast, (5) cervix, (6) colon and rectum, (7) gallbladder and biliary tract, (8) Hodgkin lymphoma, (9) kidney and renal pelvis, (10) leukaemia, (11) liver and intrahepatic bile duct, (12) lung and bronchus, (13) melanoma, (14) multiple myeloma, (15) non-Hodgkin lymphoma, (16) oesophagus, (17) oropharynx, (18) ovary, (19) pancreas, (20) prostate, (21) small intestine, (22) spinal cord and nervous system, (23) stomach, (24) testis, (25) thyroid, and (26) uterus. For analyses on late morbidities, we considered 144 health conditions where observations were coded using Read, SNOMED and ICD-10 terminology. We utilised open-access electronic health record phenotypes (https://phenotypes.healthdatagateway.org) that had been validated^3,18,31–33^. The 144 conditions were classified into 12 organ systems. We considered seven cancer treatment variables: (1) surgery only (including those of unknown type, which may include transplants), (2) radiotherapy only, (3) systemic anti-cancer treatment only (hereafter referred to as chemotherapy), (4) chemotherapy and surgery, (5) chemotherapy and radiotherapy, (6) radiotherapy and surgery, and (7) chemotherapy, radiotherapy and surgery. We considered 10 types of chemotherapy drug variables: (1) alkylating agents, (2) anthracyclines, (3) antimetabolites, (4) biological response modifiers including monoclonal antibodies and immunotherapies, (5) hormonal agents (including corticosteroid hormones and sex hormones), (6) kinase inhibitors, (7) non-anthracycline antitumour antibiotics, (8) plant alkaloids and natural products (excluding vinca alkaloids), (9) platinum agents, and (10) vinca alkaloids.

### Statistical analyses

To capture the burden of recurrent events, cumulative burdens of health conditions were identified using the validated mean cumulative count (MCC) method^27^. We employed the cumulative burden method rather than cumulative incidence because the latter only considers the first occurrence of an event in each person. Cumulative burden was estimated after accounting for competing risks, where death was considered as a competing risk event as it precludes the occurrence of other events. Cumulative burden is interpreted as the mean count of events per 100 individuals within a specific population at a given time, meaning that it can adopt any positive number. For example, cumulative burden of 5 by age 60 means that there is an average of 5 events occurring per 100 individuals at age 60. We calculated 95% confidence intervals using the bootstrap percentile method^27^. Following a previous methodology^19^, each condition was assigned to one of the three event subtypes: (1) single, recurrent, (2) chronic, non-recurrent, and (3) chronic, recurrent. Single, recurrent events were events that can occur multiple times (e.g., acute kidney injury)—all events were counted using the dates of onset. Chronic, non-recurrent events were events that are ongoing that could not happen recurrently (e.g., fatty liver disease). Chronic, non-recurrent events were counted only once at the time of onset. Chronic, recurrent events were the hybrid of the two prior subtypes (e.g., dilated cardiomyopathy). We did not employ the common terminology criteria for adverse events in estimating the cumulative burden and have not considered the severity of conditions.

Given that a vast amount of cumulative burden data was generated in this study, in addition to reporting the mean cumulative count values and 95% confidence intervals, we calculated fold difference to highlight conditions that had large differences in cumulative burden between survivors and controls. Fold difference was calculated as the cumulative burden in survivors versus controls. For example, if the cumulative burden in survivors for a particular condition is 5 events per 100 individuals and the cumulative burden in controls is 1 event per 100 individuals, then, the fold difference is 5/1 = 5 (five times higher in survivors compared with controls). For conditions where controls had a higher cumulative burden (e.g., 1 event in survivors versus 3 events in controls), then the fold difference is expressed as 3/1 = 3 (three times higher in controls compared with survivors). Where there were no events in either survivors or controls fold difference is not calculated to avoid a divisor of zero.

Logistic regression was performed to determine the association between treatment exposures and diagnoses of health conditions. Regression models were adjusted for age at cancer diagnosis, cancer type, sex and socioeconomic deprivation status. Excess years of life lost (YLL) were estimated using the lillies^34^ package in R, which had been validated by other studies^35–37^. Excess YLL is defined as the difference in YLL between survivors who developed a health condition minus survivors who did not develop a health condition.

All analyses were performed using R studio (v3.6.3). The following packages were used: tidyverse (v1.3.2), tableone (v0.13.0), etm (v1.1.1), mstate (v0.3.2), cmprsk (v2.2.11), lillies (v0.2.9), reshape (v0.8.9), splines (v0.4.5), survival (v3.3), survminer (v0.4.9), matchit (v4.3.4), DataCombine (v0.2.21), and data.table (v1.14.6).

### Reporting summary

Further information on research design is available in the Nature Portfolio Reporting Summary linked to this article.

## Supplementary information

Supplementary Information

Description of Additional Supplementary Files

Supplementary Data 1-15

Reporting Summary

## Data Availability

This study employs patient data in England collected as part of their care and support. The data is subject to controlled access and reason for controlled access is as follows: since electronic health records are classified as sensitive data by the UK Data Protection Act, information governance restrictions are in place to protect patient confidentiality and prevent data sharing in public repositories. Data access is conditioned on successful ethics application to the Medicines and Healthcare products Regulatory Agency and assessment by the Independent Scientific Advisory Committee. All summarised data and results are provided as a Source Data file and further enquiries can be directed to the lead author Wai Hoong Chang (wai.chang@ucl.ac.uk) who will aim to respond to requests within 2 weeks.

## Code availability

All analyses were performed using R Studio (v3.6.3) within an airlocked research environment using analysis codes retrieved from this website (https://sites.ualberta.ca/~yyasui/software.html).

## References

[1] Marzorati C, Riva S, Pravettoni G (2017). Who is a cancer survivor? A systematic review of published definitions. J. Cancer Educ..

[2] European Cancer Patient Coalition. ESMO Patient Guide on Survivorship. https://ecpc.org/policy/survivorship/.

[3] Denaxas S (2019). UK phenomics platform for developing and validating electronic health record phenotypes: CALIBER. J. Am. Med. Inform. Assoc..

[4] Lai AG (2021). An informatics consult approach for generating clinical evidence for treatment decisions. BMC Med. Inform. Decis. Mak..

[5] National Cancer Registration and Analysis Service. NCRAS Publications. http://www.ncin.org.uk/publications/.

[6] Sturgeon KM (2019). A population-based study of cardiovascular disease mortality risk in US cancer patients. Eur. Heart J..

[7] Sima CS, Panageas KS, Schrag D (2010). Cancer screening among patients with advanced cancer. Jama.

[8] Tini G (2019). Arterial hypertension in cancer: the elephant in the room. Int. J. Cardiol..

[9] Blumenthal JA (2021). Effects of lifestyle modification on patients with resistant hypertension: results of the TRIUMPH randomized clinical trial. Circulation.

[10] Morote J (2007). Prevalence of osteoporosis during long-term androgen deprivation therapy in patients with prostate cancer. Urology.

[11] Pagani O (2014). Adjuvant exemestane with ovarian suppression in premenopausal breast cancer. N. Engl. J. Med..

[12] Rabaglio M (2009). Bone fractures among postmenopausal patients with endocrine-responsive early breast cancer treated with 5 years of letrozole or tamoxifen in the BIG 1-98 trial. Ann. Oncol..

[13] Saarto T (1997). Chemical castration induced by adjuvant cyclophosphamide, methotrexate, and fluorouracil chemotherapy causes rapid bone loss that is reduced by clodronate: a randomized study in premenopausal breast cancer patients. J. Clin. Oncol..

[14] Costa S, Reagan MR (2019). Therapeutic irradiation: consequences for bone and bone marrow adipose tissue. Front. Endocrinol..

[15] Gnant MF (2007). Zoledronic acid effectively prevents cancer treatment-induced bone loss in premenopausal women receiving adjuvant endocrine therapy for hormone-responsive breast cancer: a report from the Austrian Breast and Colorectal Cancer Study Group. J. Clin. Oncol..

[16] Maddams J, Utley M, Møller H (2012). Projections of cancer prevalence in the United Kingdom, 2010-−2040. Br. J. Cancer.

[17] Bhakta N (2017). The cumulative burden of surviving childhood cancer: an initial report from the St Jude Lifetime Cohort Study (SJLIFE). Lancet.

[18] Chang WH (2021). Late effects of cancer in children, teenagers and young adults: population-based study on the burden of 183 conditions, in-patient and critical care admissions and years of life lost. Lancet Regional Health Eur..

[19] Bhakta N (2016). Cumulative burden of cardiovascular morbidity in paediatric, adolescent, and young adult survivors of Hodgkin’s lymphoma: an analysis from the St Jude Lifetime Cohort Study. lancet Oncol..

[20] Department of Health and Social Care. Living With and Beyond Cancer: Taking Action to Improve Outcomes. https://www.gov.uk/government/publications/living-with-and-beyond-cancer-taking-action-to-improve-outcomes.

[21] Lewis RA (2009). Follow-up of cancer in primary care versus secondary care: systematic review. Br. J. Gen. Pract..

[22] National Institute for Health and Care Excellence. Prostate cancer: diagnosis and management. https://www.nice.org.uk/guidance/ng131/chapter/Recommendations. 31393679

[23] Stegmann ME (2019). Correspondence between primary and secondary care about patients with cancer: a qualitative mixed-methods analysis. Eur. J. Cancer Care.

[24] Gilchrist SC (2019). Circulation.

[25] Armenian SH (2017). Prevention and monitoring of cardiac dysfunction in survivors of adult cancers: American Society of Clinical Oncology Clinical Practice Guideline. J. Clin. Oncol..

[26] Miguel TP (2020). Chemoradiation for cervical cancer treatment portends high risk of pelvic floor dysfunction. PLoS ONE.

[27] Dong H (2015). Estimating the burden of recurrent events in the presence of competing risks: The method of mean cumulative count. Am. J. Epidemiol..

[28] American Cancer Society. Cancer treatment and survivorship facts and figures 2019–2021. *Am. Cancer Soc.* 1–48 (2019).

[29] Alfano CM (2019). Equitably improving outcomes for cancer survivors and supporting caregivers: a blueprint for care delivery, research, education, and policy. Cancer J. Clin..

[30] Murphy CC (2018). Polypharmacy and patterns of prescription medication use among cancer survivors. Cancer.

[31] Kuan V (2019). A chronological map of 308 physical and mental health conditions from 4 million individuals in the English National Health Service. Lancet Digital Health.

[32] Lai AG (2020). Estimated impact of the COVID-19 pandemic on cancer services and excess 1-year mortality in people with cancer and multimorbidity: near real-time data on cancer care, cancer deaths and a population-based cohort study. BMJ Open.

[33] Chang WH, Mueller SH, Chung S-C, Foster GR, Lai AG (2022). Increased burden of cardiovascular disease in people with liver disease: unequal geographical variations, risk factors and excess years of life lost. J. Transl. Med..

[34] Plana-Ripoll O (2020). lillies: An R package for the estimation of excess Life Years Lost among patients with a given disease or condition. PLoS ONE.

[35] Erlangsen A (2017). Cause-specific life-years lost in people with mental disorders: a nationwide, register-based cohort study. Lancet Psychiatry.

[36] Plana-Ripoll O (2019). A comprehensive analysis of mortality-related health metrics associated with mental disorders: a nationwide, register-based cohort study. Lancet.

[37] Laursen TM (2019). Cause-specific life years lost among persons diagnosed with schizophrenia: is it getting better or worse?. Schizophr. Res..

