# Supplementary material for: Cumulative burden of 144 conditions, critical care hospitalisation and premature mortality across 26 adult cancers
Source: Nat Commun. 2023 Mar 17;14:1484. doi: 10.1038/s41467-023-37231-3 (PMC10023774; doi:10.1038/s41467-023-37231-3)

Supplementary Figure 1. Flow diagram of survivors and controls.

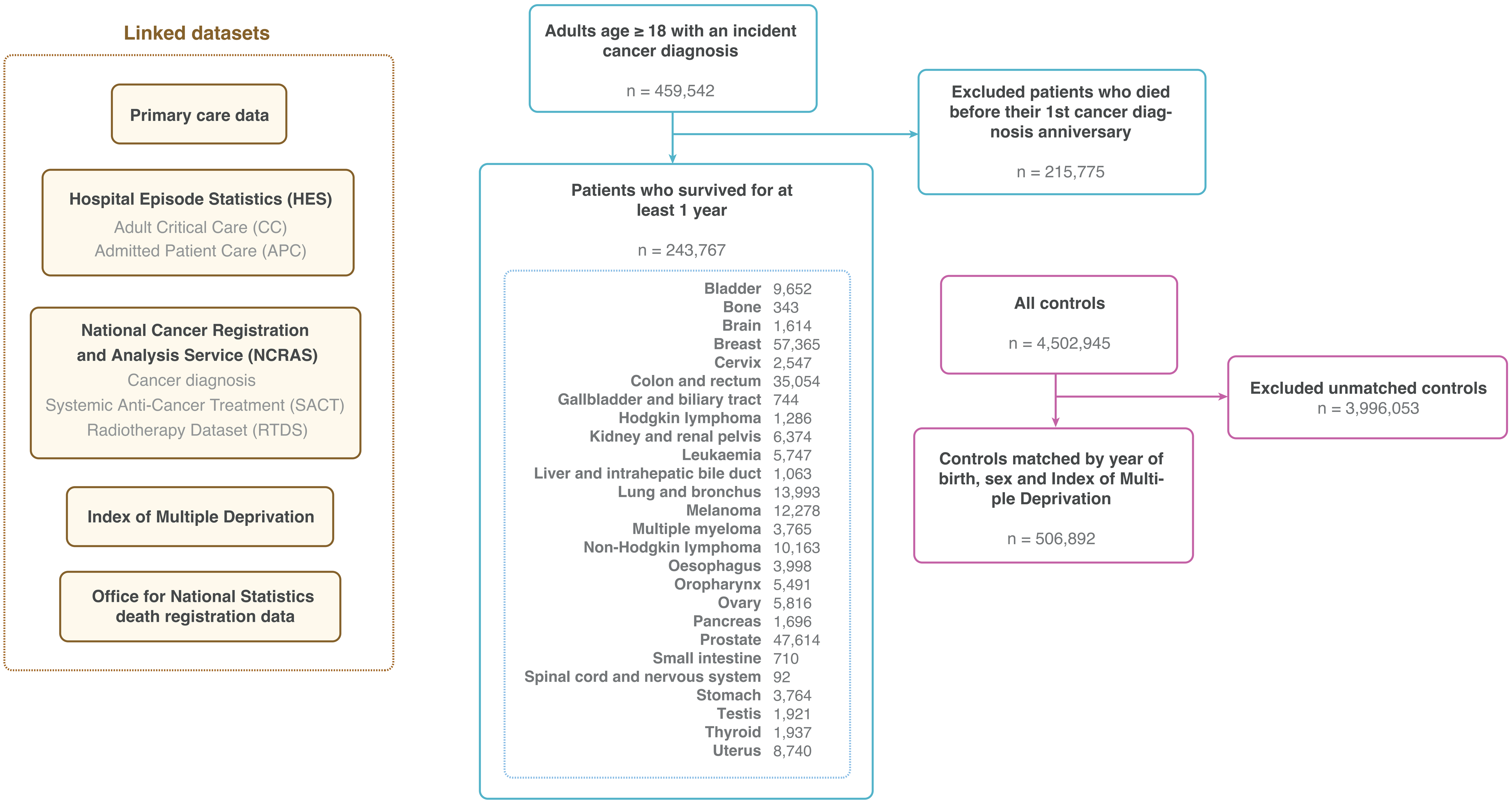

Supplementary Figure 2. Cumulative burden of health conditions for cancer survivors and controls from the lowest socioeconomic group based on the Index of Multiple Deprivation (IMD). Health conditions were rank ordered according to the fold difference at age 60 in individuals from the lowest socioeconomic group (see corresponding results for the highest socioeconomic group in Figure 1).

(A) Lollipop graphs depict the fold difference of cumulative burden in survivors versus controls for different age groups. The fold difference is annotated in each lollipop. Conditions that have a higher cumulative burden in survivors are shown on the right side within each age-specific plot, while conditions with a higher cumulative burden in controls are shown on the left. Where there are no events in either survivors or controls, the fold difference is not calculated.

(B) Heatmaps depict cumulative burden counts per 100 individuals for survivors and controls. Each tile in the heatmap corresponds to cumulative burden count per 100 persons for each condition-specific outcome at different age groups. For example, a cumulative burden of 6.13 for left bundle branch block in controls at age 80 corresponds to 6.13 events per 100 individuals. Cumulative burden values were separated into 10-quantiles (10 groups) resulting in quantile colour representation of the heatmaps. Source data are provided as a Source Data file.

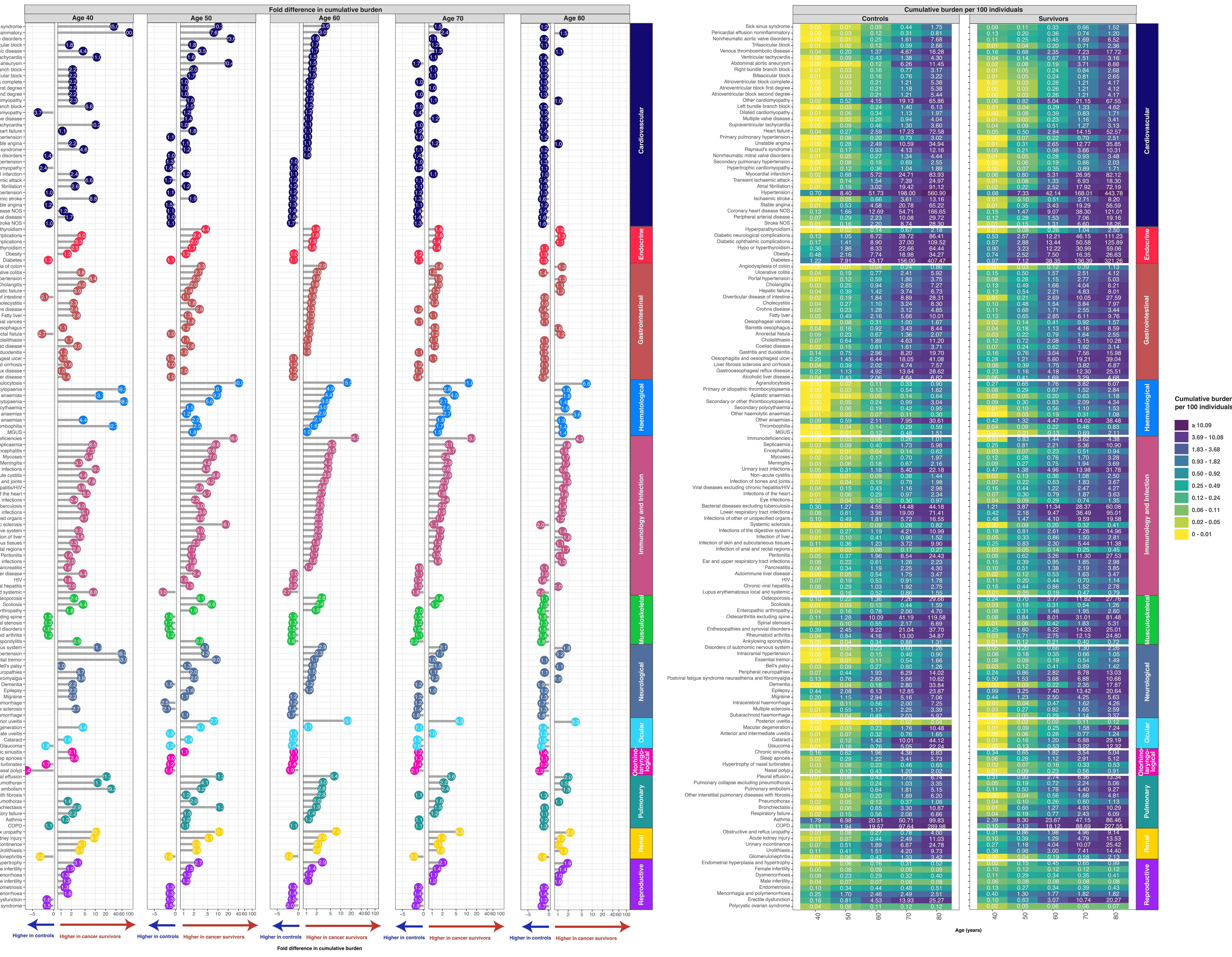

Supplementary Figure 3. Circular dendrogram for bladder cancer depicting the fold difference of cumulative burden in survivors versus controls at age 60 where conditions with a fold difference of ≥ 2 are shown. The area of the nodes is proportional to the fold-difference of each condition, and the conditions are ranked from the highest to lowest fold difference. Source data are provided as a Source Data file.

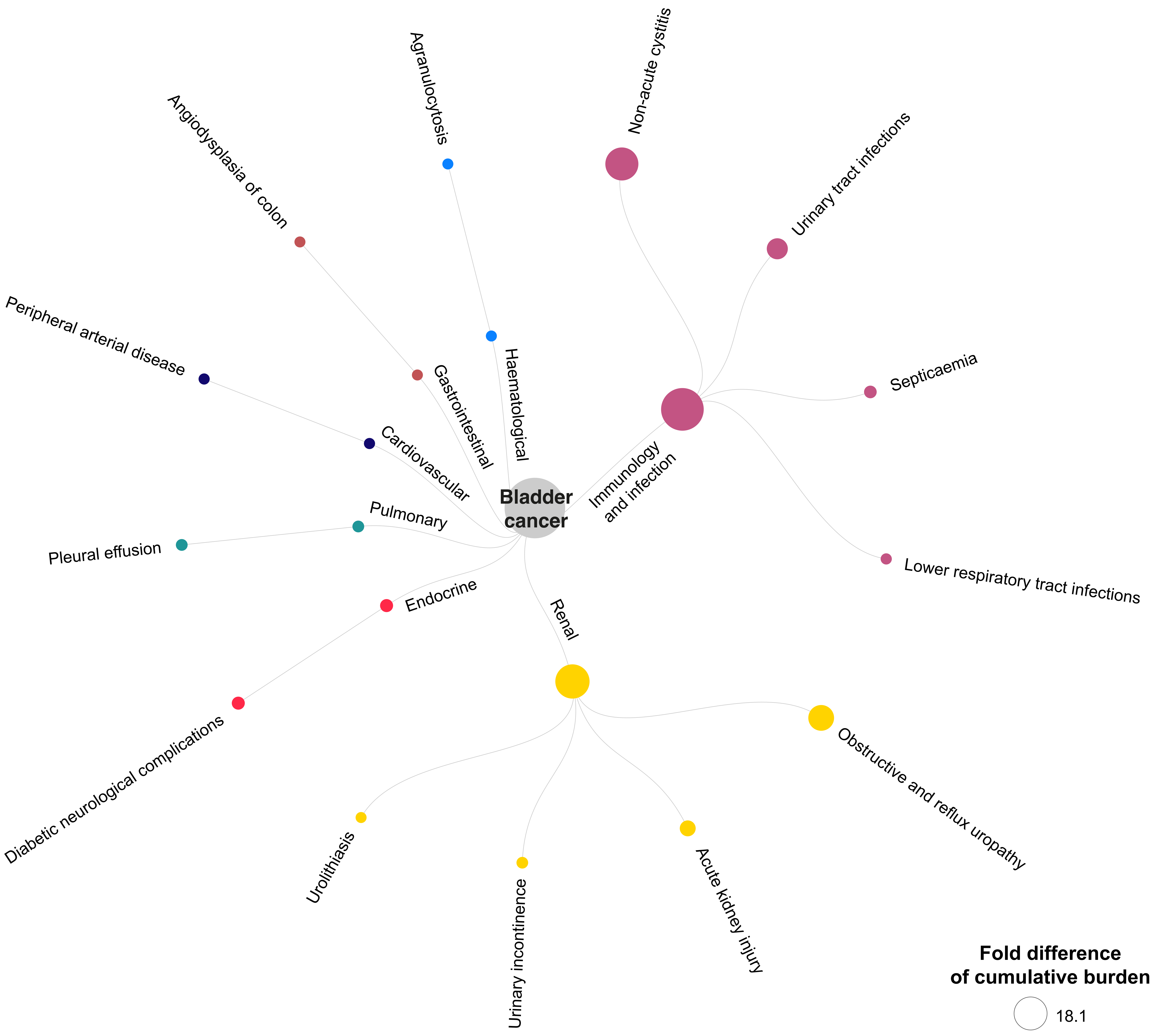

Supplementary Figure 4. Circular dendrogram for bone cancer depicting the fold difference of cumulative burden in survivors versus controls at age 60 where conditions with a fold difference of ≥ 2 are shown. The area of the nodes is proportional to the fold-difference of each condition, and the conditions are ranked from the highest to lowest fold difference. Source data are provided as a Source Data file.

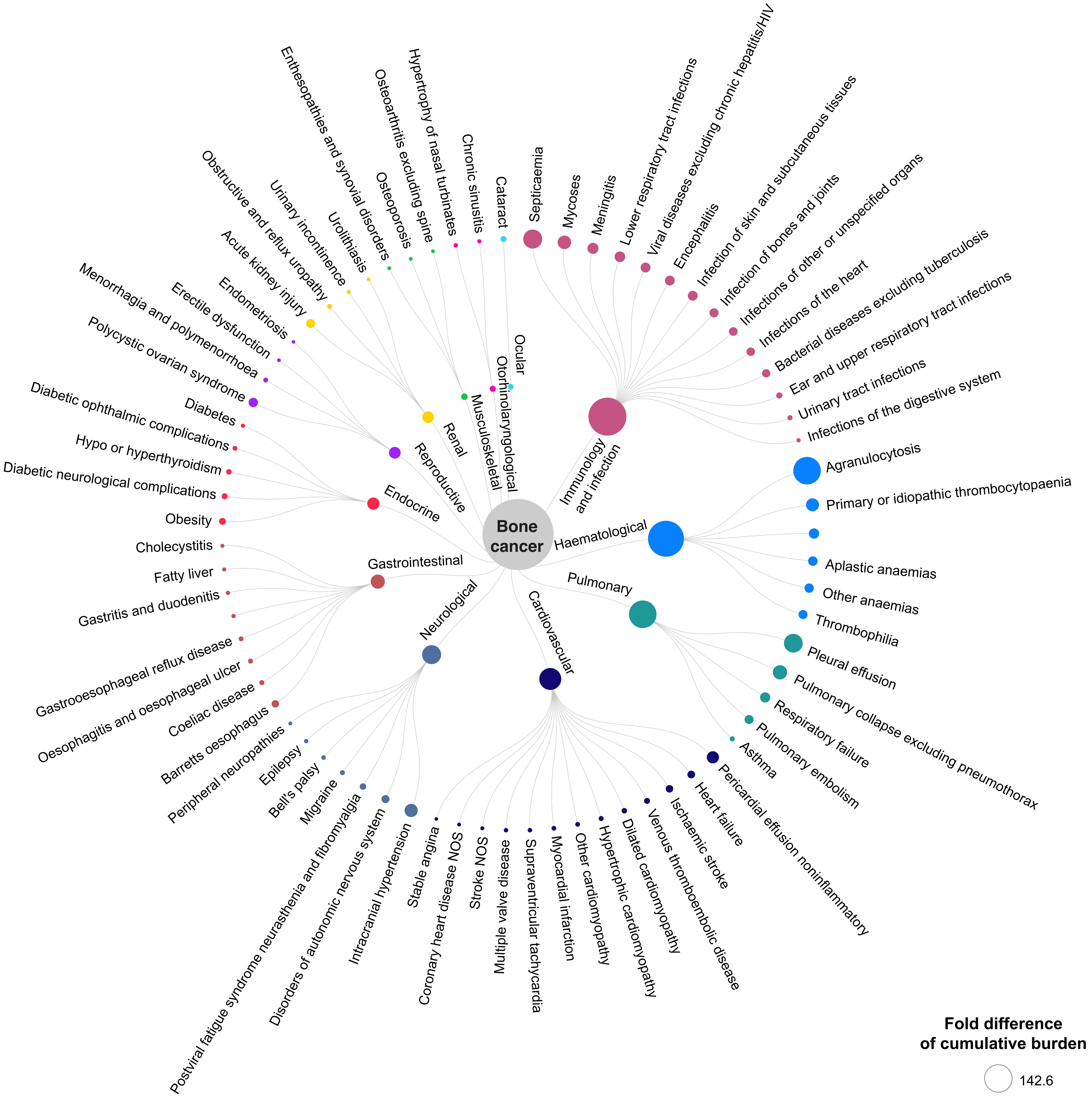

Supplementary Figure 5. Circular dendrogram for cervical cancer depicting the fold difference of cumulative burden in survivors versus controls at age 60 where conditions with a fold difference of ≥ 2 are shown. The area of the nodes is proportional to the fold-difference of each condition, and the conditions are ranked from the highest to lowest fold difference. Source data are provided as a Source Data file.

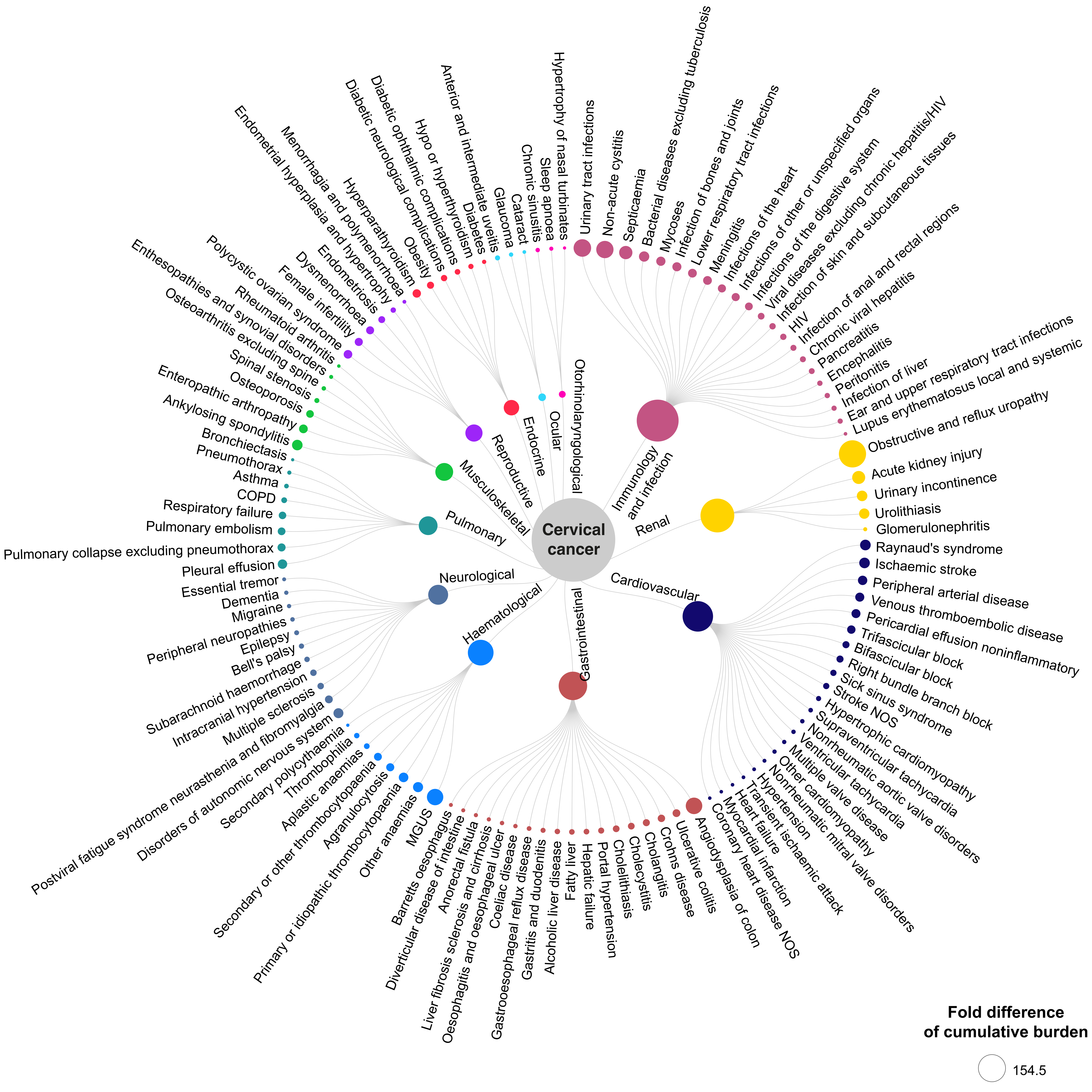

Supplementary Figure 6. Circular dendrogram for colorectal cancer depicting the fold difference of cumulative burden in survivors versus controls at age 60 where conditions with a fold difference of ≥ 2 are shown. The area of the nodes is proportional to the fold-difference of each condition, and the conditions are ranked from the highest to lowest fold difference. Source data are provided as a Source Data file.

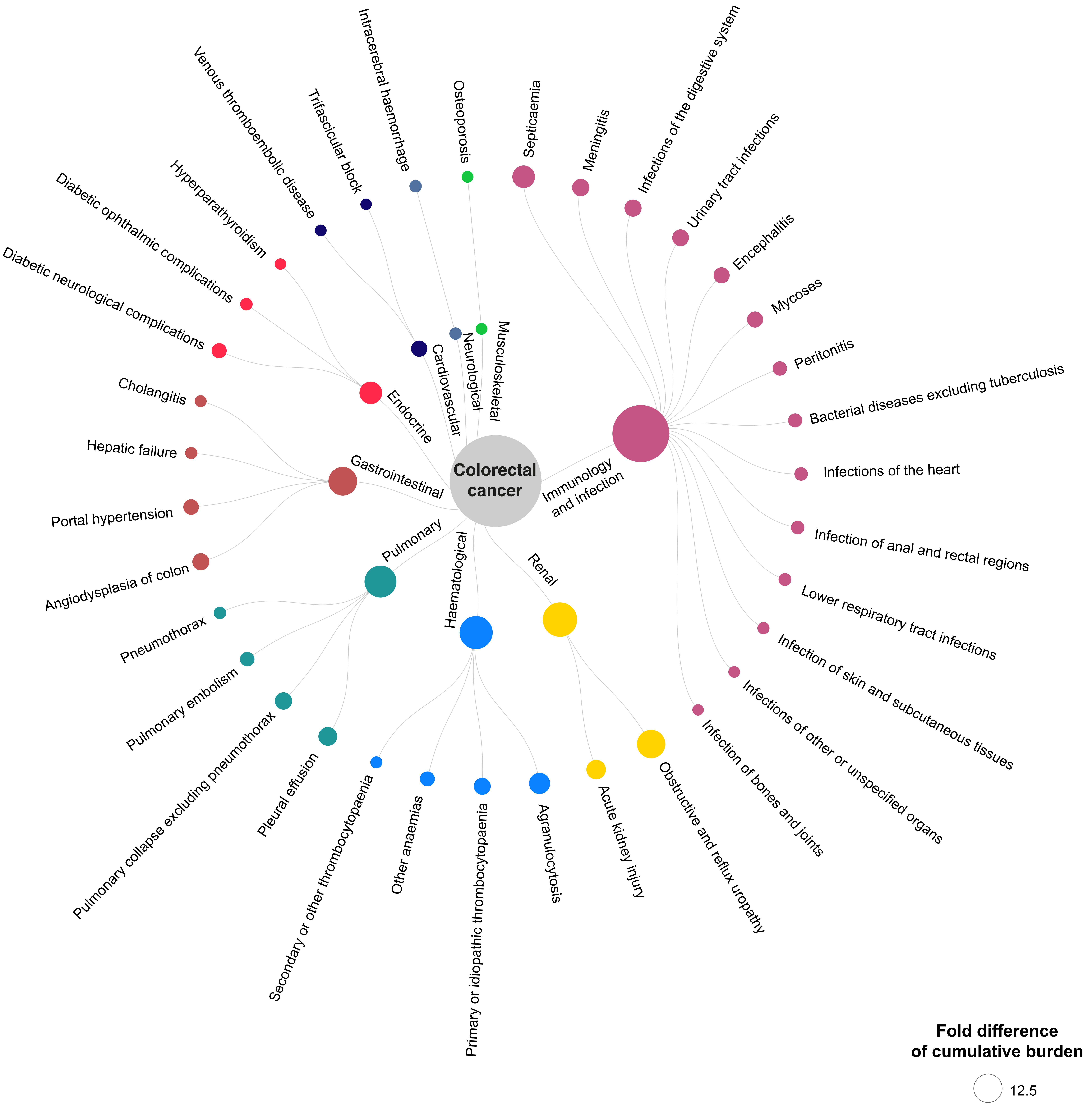

Supplementary Figure 7. Circular dendrogram for gallbladder and biliary tract cancer depicting the fold difference of cumulative burden in survivors versus controls at age 60 where conditions with a fold difference of ≥ 2 are shown. The area of the nodes is proportional to the fold-difference of each condition, and the conditions are ranked from the highest to lowest fold difference. Source data are provided as a Source Data file.

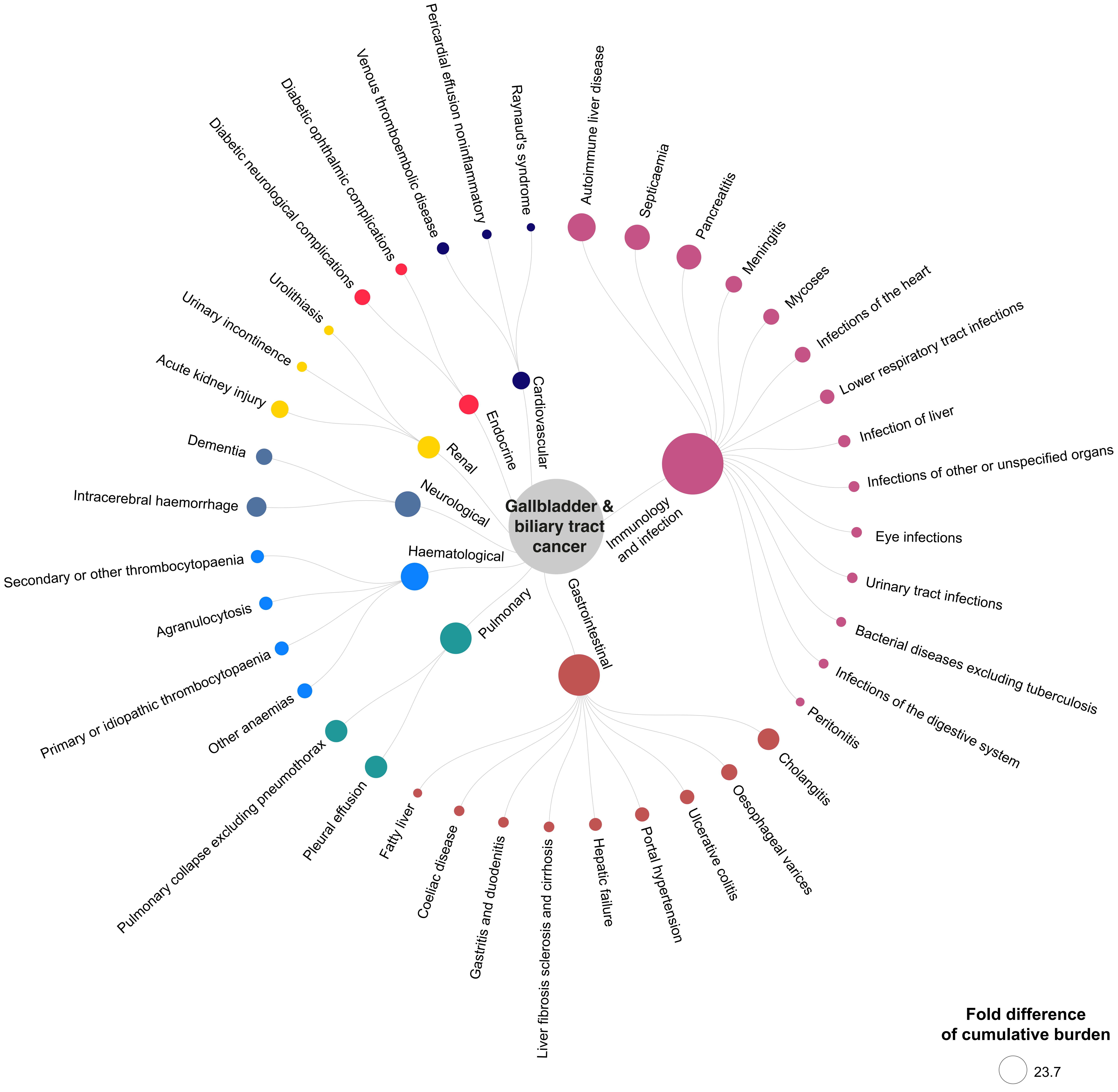

Supplementary Figure 8. Circular dendrogram for Hodgkin lymphoma depicting the fold difference of cumulative burden in survivors versus controls at age 60 where conditions with a fold difference of ≥ 2 are shown. The area of the nodes is proportional to the fold-difference of each condition, and the conditions are ranked from the highest to lowest fold difference. Source data are provided as a Source Data file.

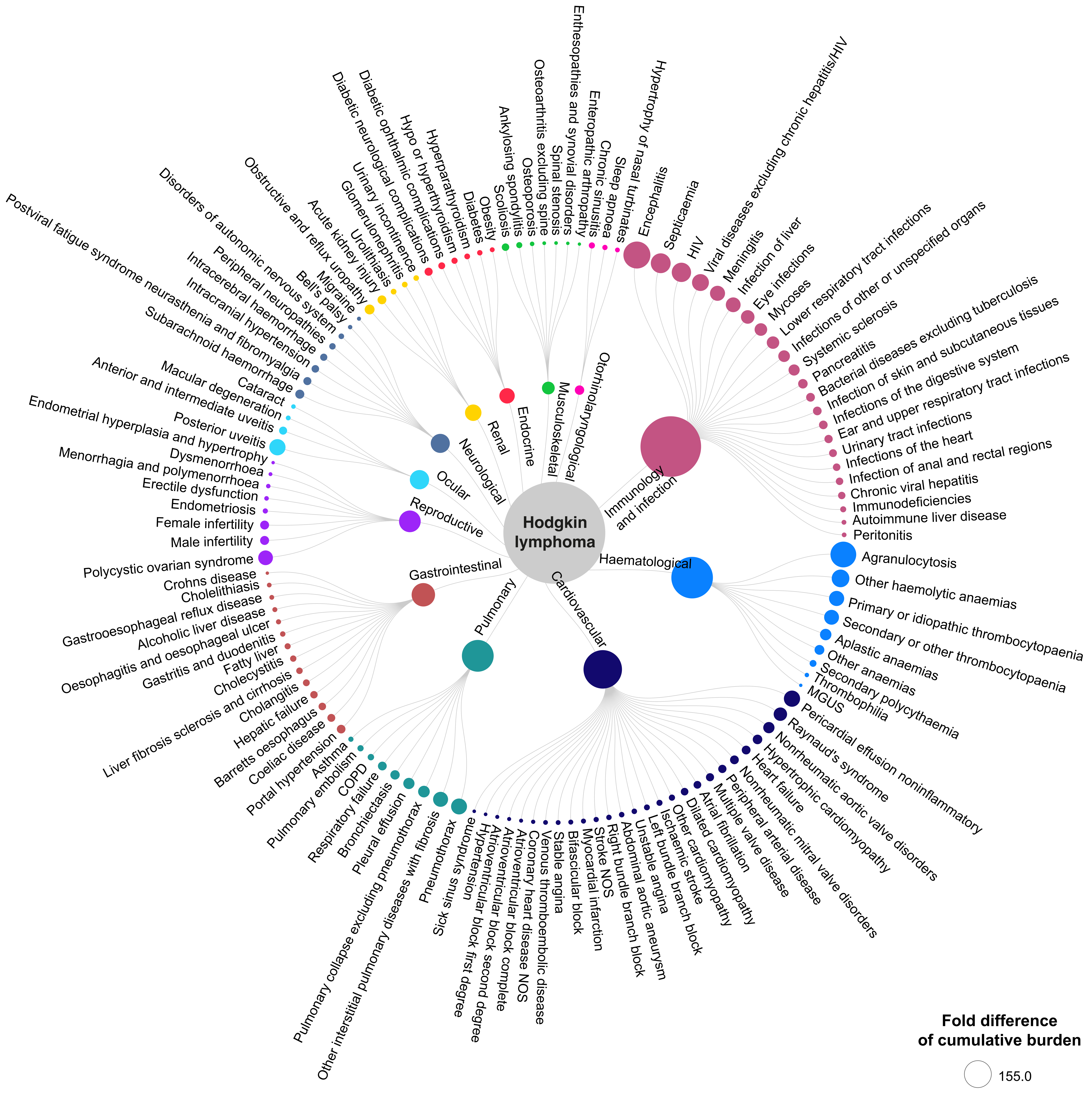

Supplementary Figure 9. Circular dendrogram for kidney cancer depicting the fold difference of cumulative burden in survivors versus controls at age 60 where conditions with a fold difference of ≥ 2 are shown. The area of the nodes is proportional to the fold-difference of each condition, and the conditions are ranked from the highest to lowest fold difference. Source data are provided as a Source Data file.

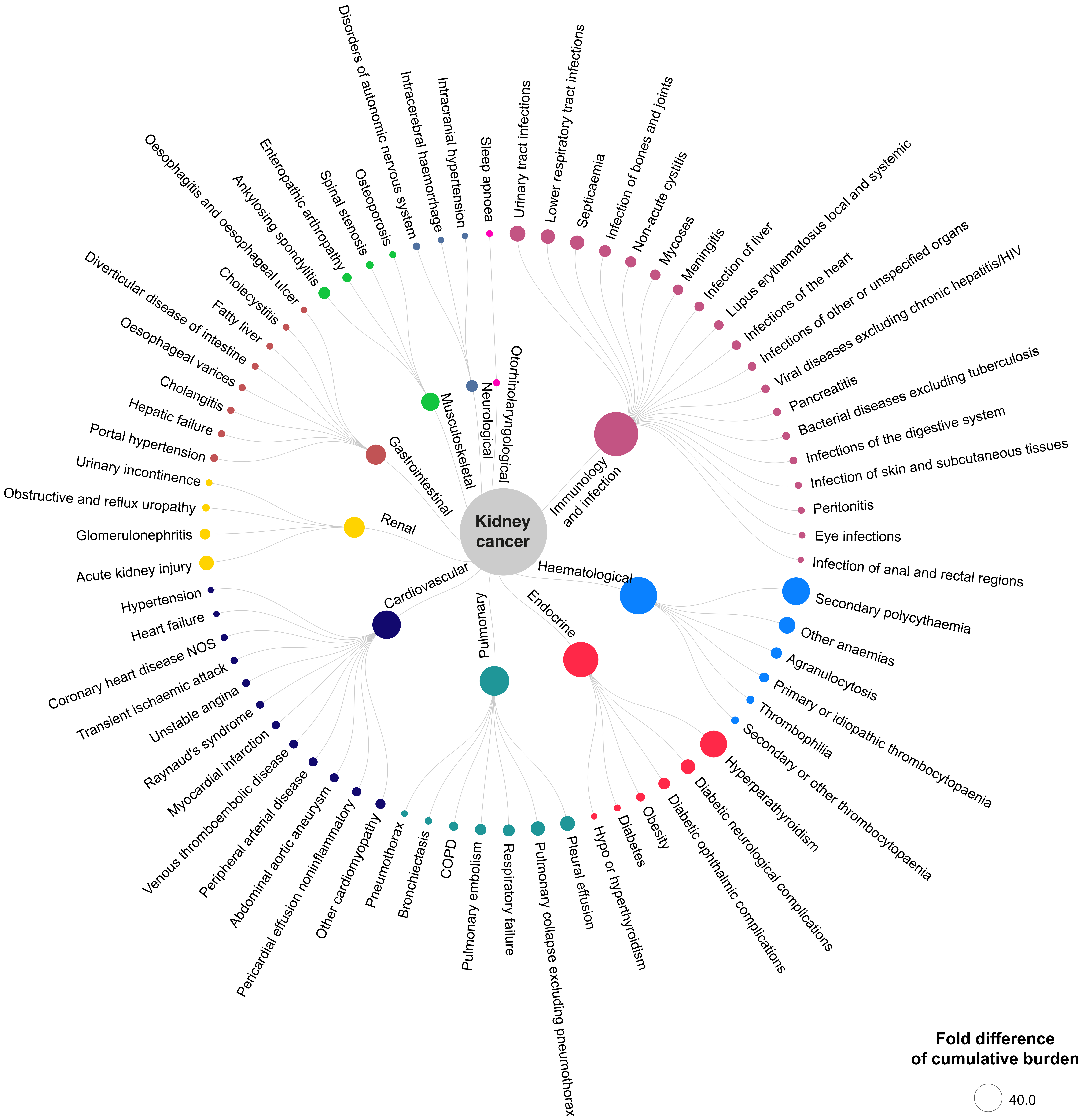

Supplementary Figure 10. Circular dendrogram for liver cancer depicting the fold difference of cumulative burden in survivors versus controls at age 60 where conditions with a fold difference of ≥ 2 are shown. The area of the nodes is proportional to the fold-difference of each condition, and the conditions are ranked from the highest to lowest fold difference. Source data are provided as a Source Data file.

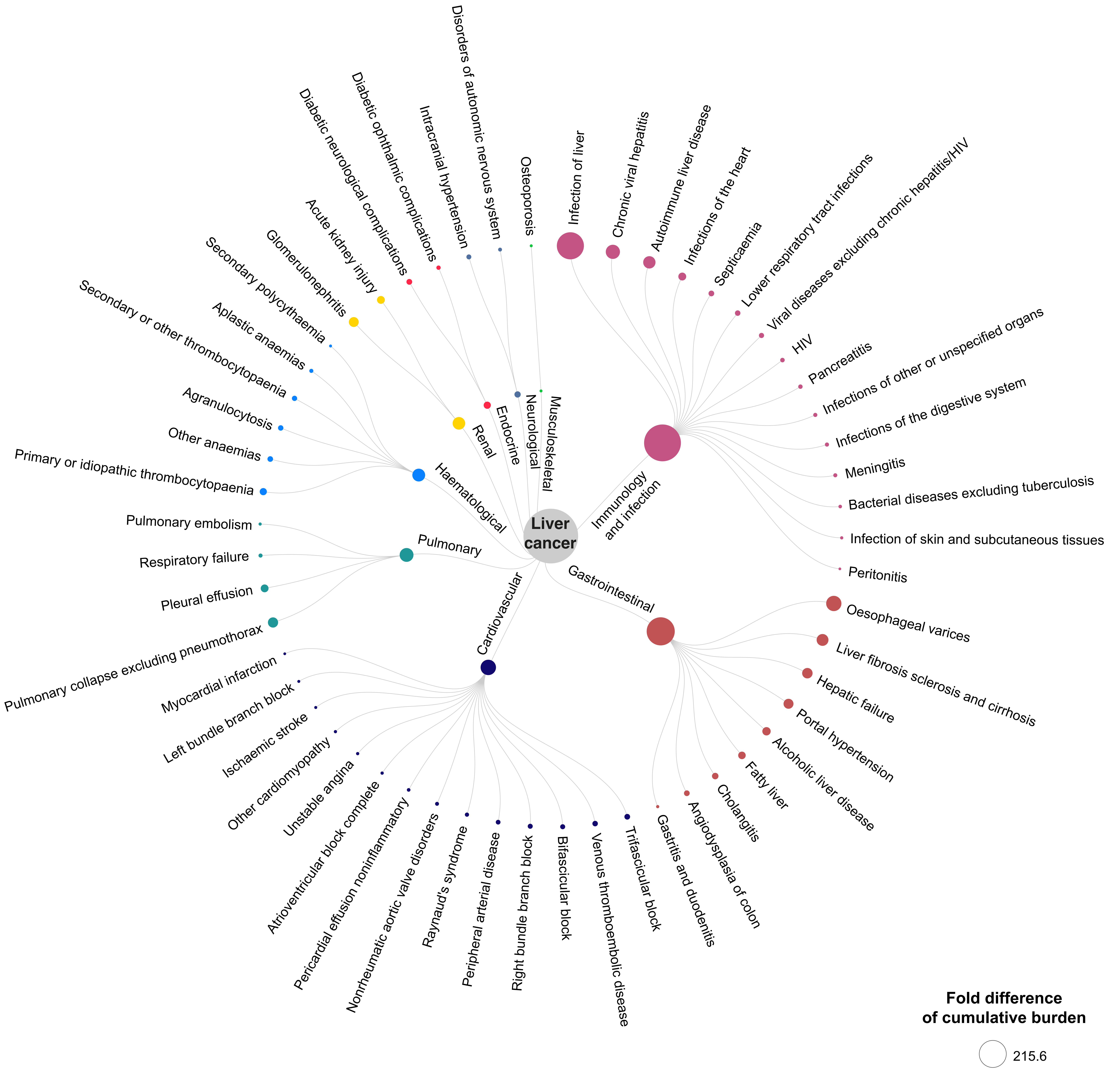

Supplementary Figure 11. Circular dendrogram for melanoma depicting the fold difference of cumulative burden in survivors versus controls at age 60 where conditions with a fold difference of ≥ 2 are shown. The area of the nodes is proportional to the fold-difference of each condition, and the conditions are ranked from the highest to lowest fold difference. Source data are provided as a Source Data file.

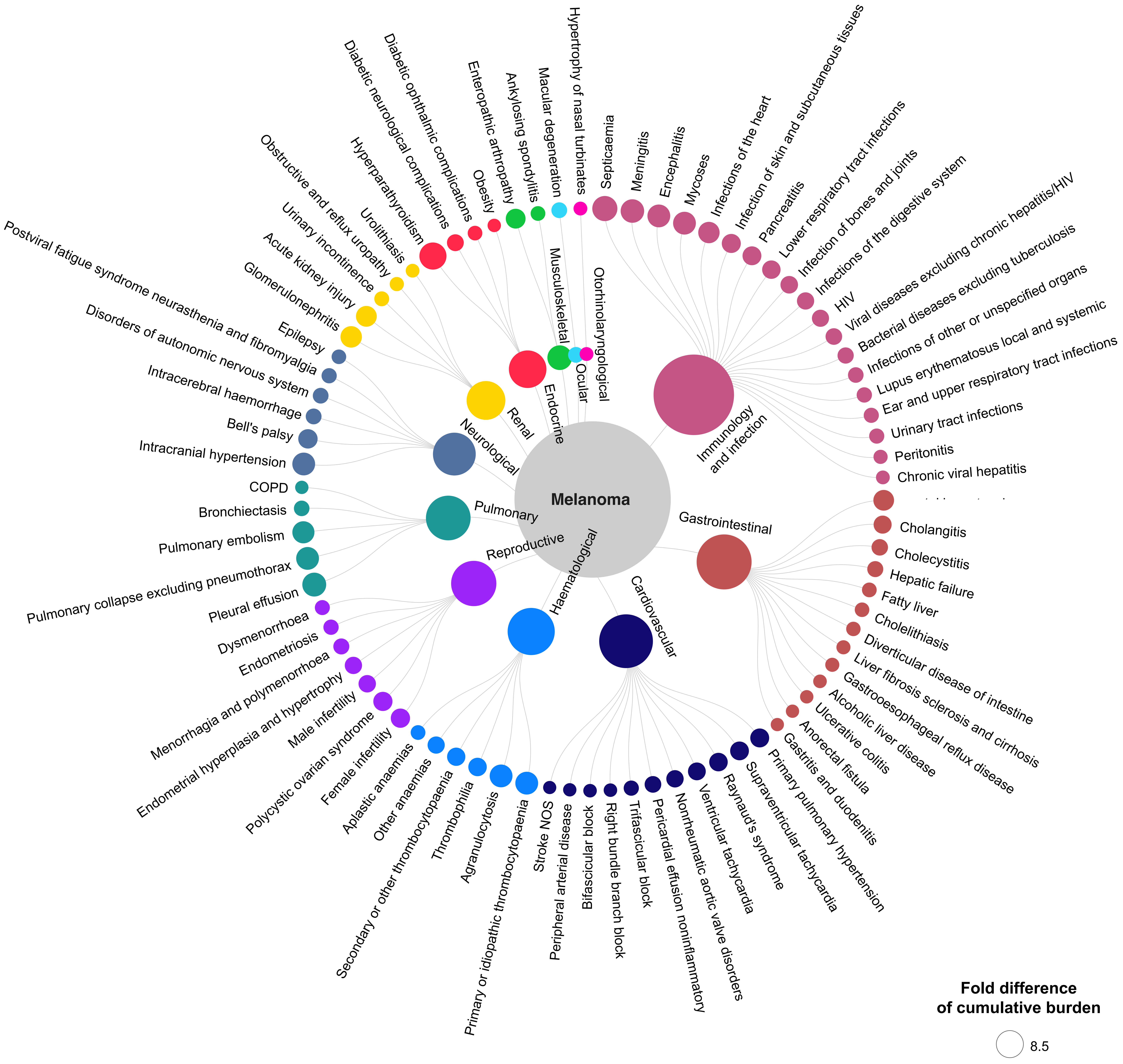

Supplementary Figure 12. Circular dendrogram for multiple myeloma depicting the fold difference of cumulative burden in survivors versus controls at age 60 where conditions with a fold difference of ≥ 2 are shown. The area of the nodes is proportional to the fold-difference of each condition, and the conditions are ranked from the highest to lowest fold difference. Source data are provided as a Source Data file.

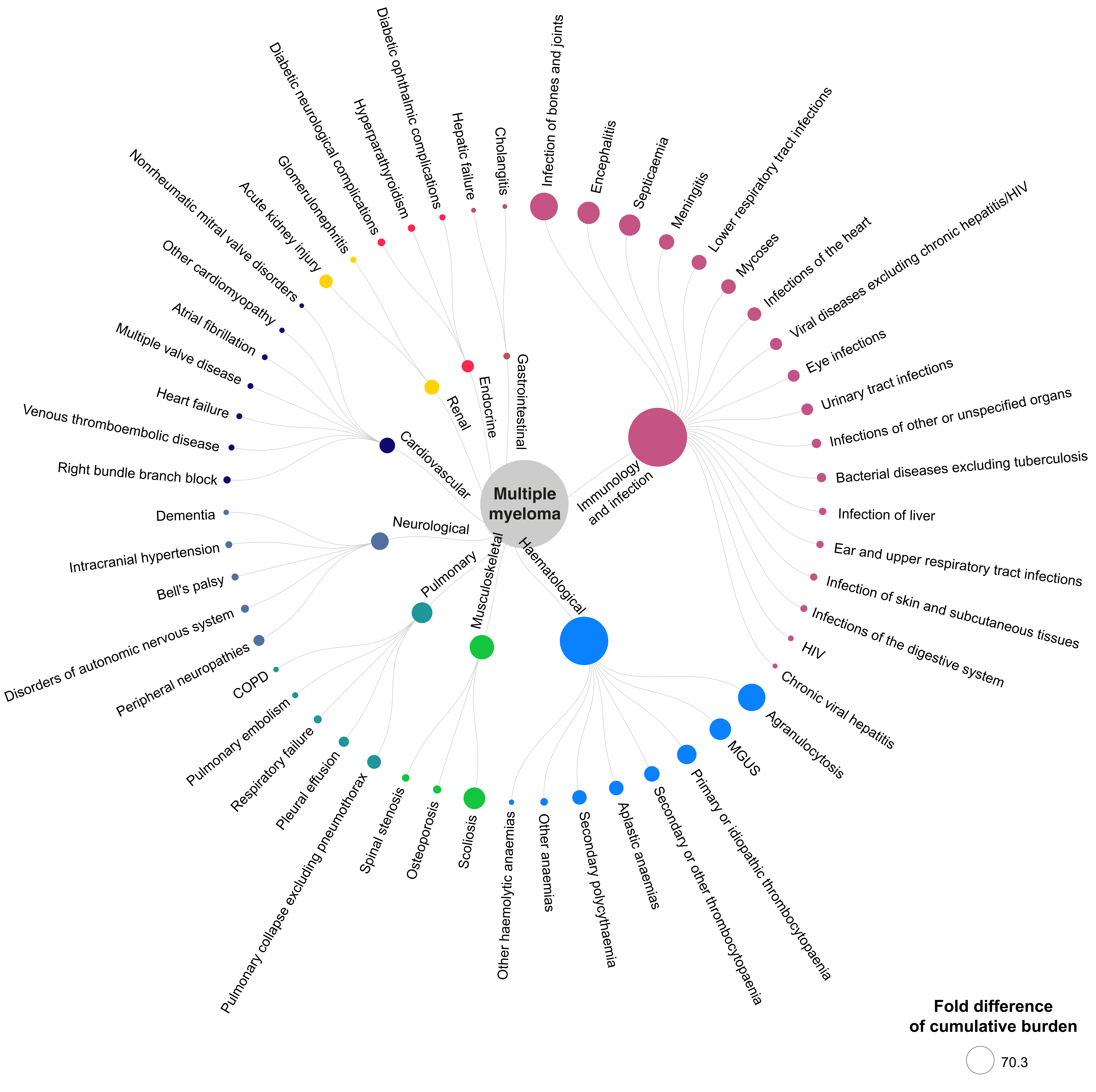

Supplementary Figure 13. Circular dendrogram for non-Hodgkin lymphoma depicting the fold difference of cumulative burden in survivors versus controls at age 60 where conditions with a fold difference of ≥ 2 are shown. The area of the nodes is proportional to the fold-difference of each condition, and the conditions are ranked from the highest to lowest fold difference. Source data are provided as a Source Data file.

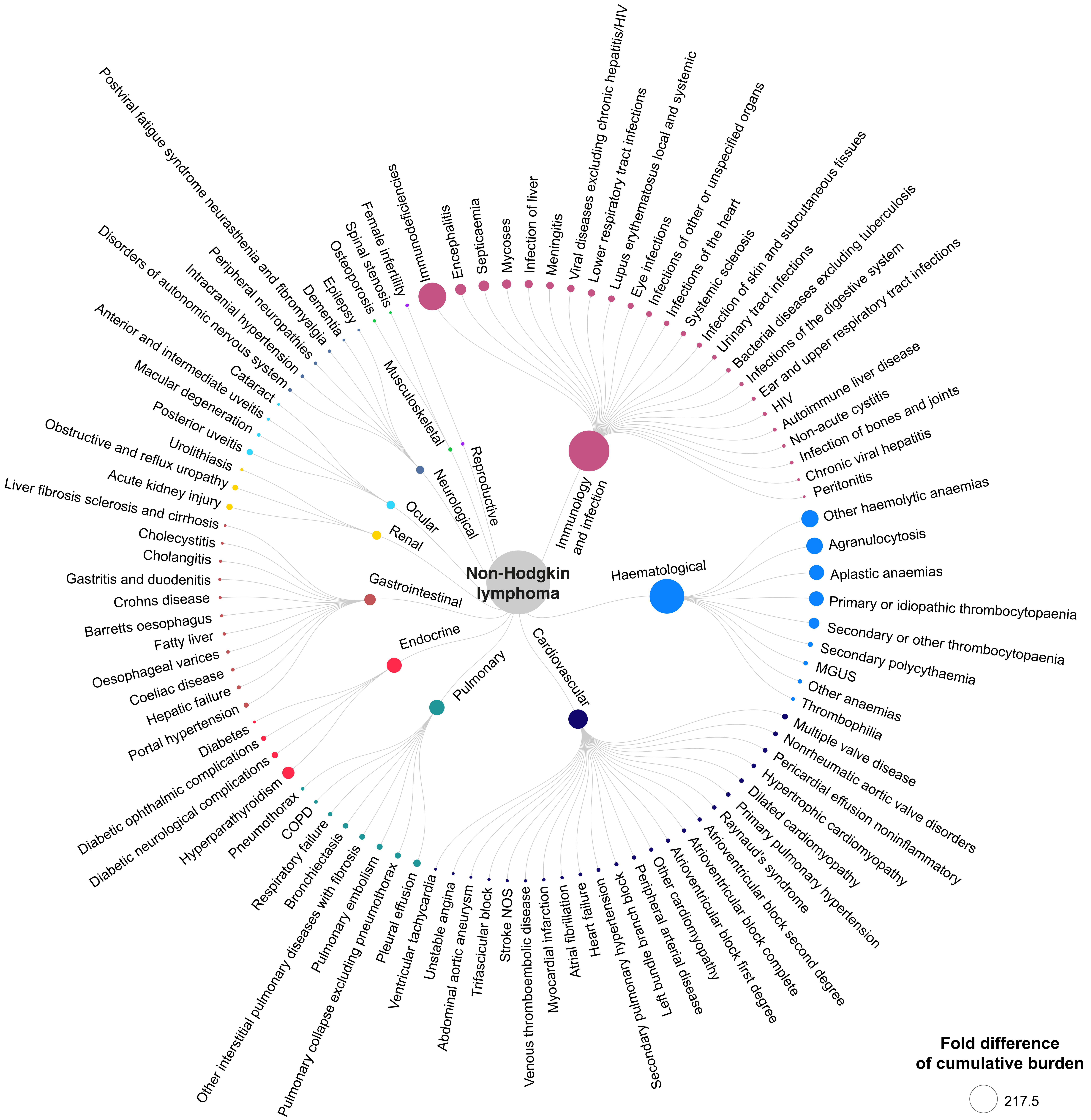

Supplementary Figure 14. Circular dendrogram for oesophageal cancer depicting the fold difference of cumulative burden in survivors versus controls at age 60 where conditions with a fold difference of ≥ 2 are shown. The area of the nodes is proportional to the fold-difference of each condition, and the conditions are ranked from the highest to lowest fold difference. Source data are provided as a Source Data file.

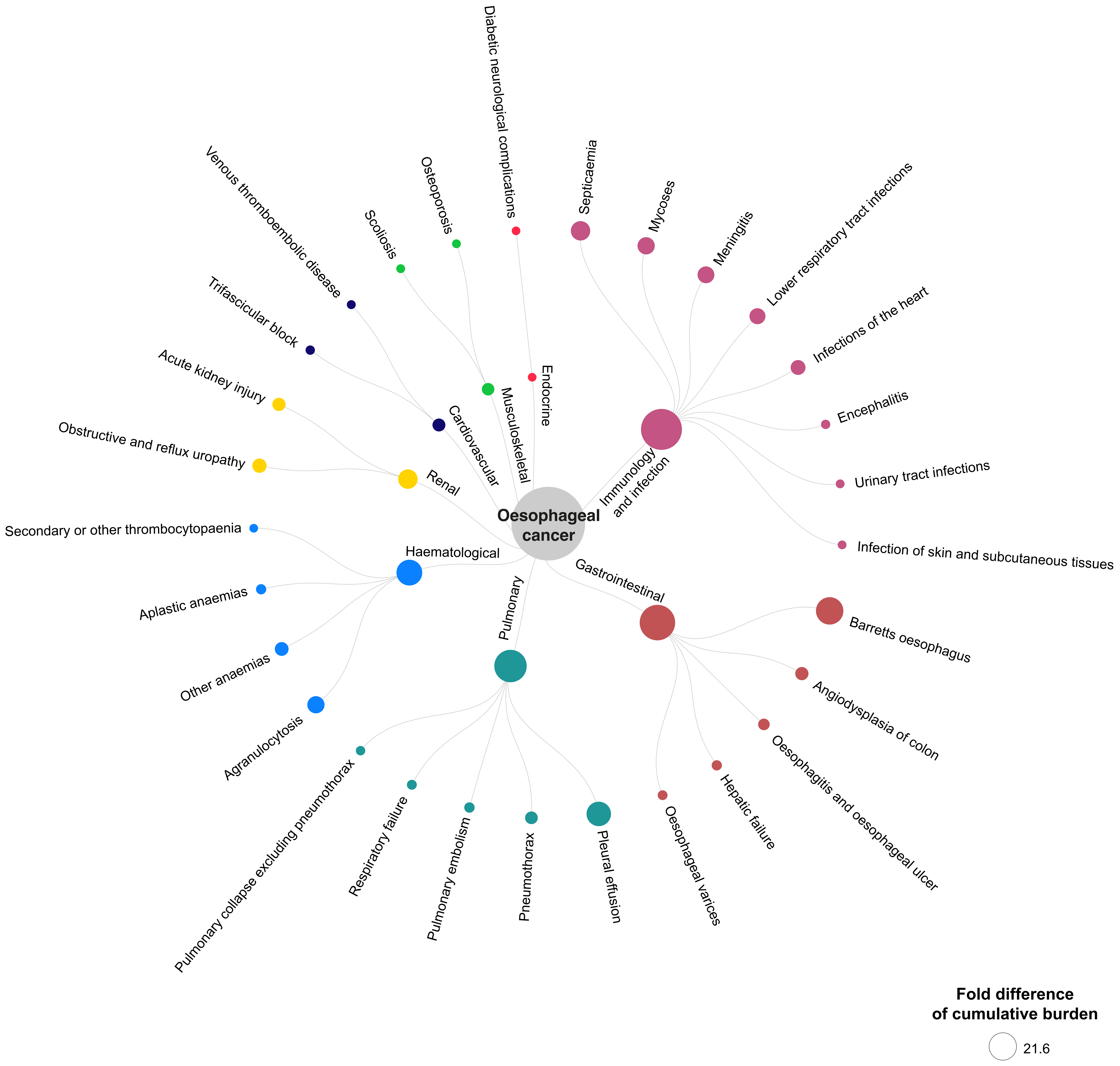

Supplementary Figure 15. Circular dendrogram for oropharyngeal cancer depicting the fold difference of cumulative burden in survivors versus controls at age 60 where conditions with a fold difference of ≥ 2 are shown. The area of the nodes is proportional to the fold-difference of each condition, and the conditions are ranked from the highest to lowest fold difference. Source data are provided as a Source Data file.

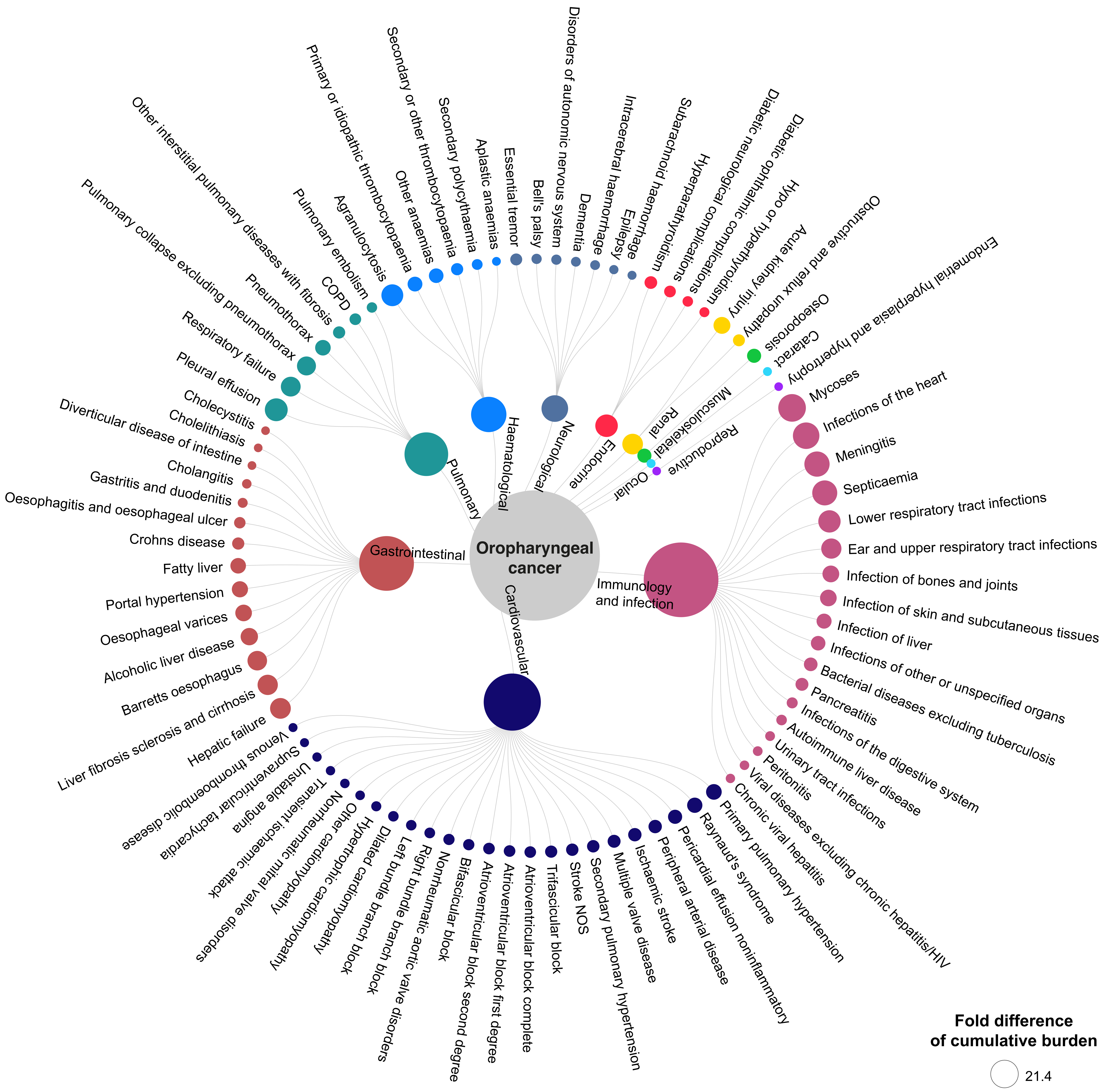

Supplementary Figure 16. Circular dendrogram for ovarian cancer depicting the fold difference of cumulative burden in survivors versus controls at age 60 where conditions with a fold difference of ≥ 2 are shown. The area of the nodes is proportional to the fold-difference of each condition, and the conditions are ranked from the highest to lowest fold difference. Source data are provided as a Source Data file.

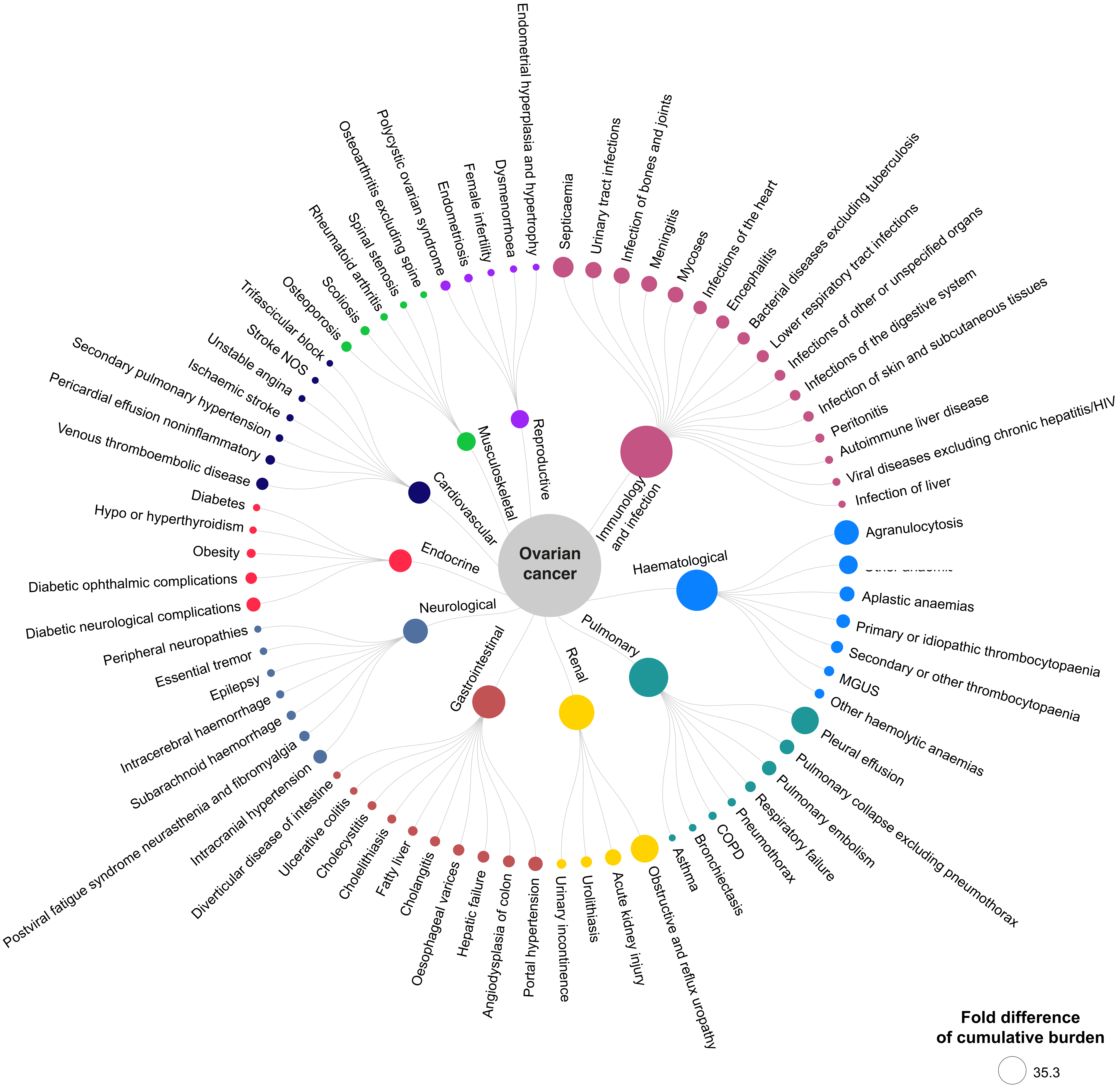

Supplementary Figure 17. Circular dendrogram for pancreatic cancer depicting the fold difference of cumulative burden in survivors versus controls at age 60 where conditions with a fold difference of ≥ 2 are shown. The area of the nodes is proportional to the fold-difference of each condition, and the conditions are ranked from the highest to lowest fold difference. Source data are provided as a Source Data file.

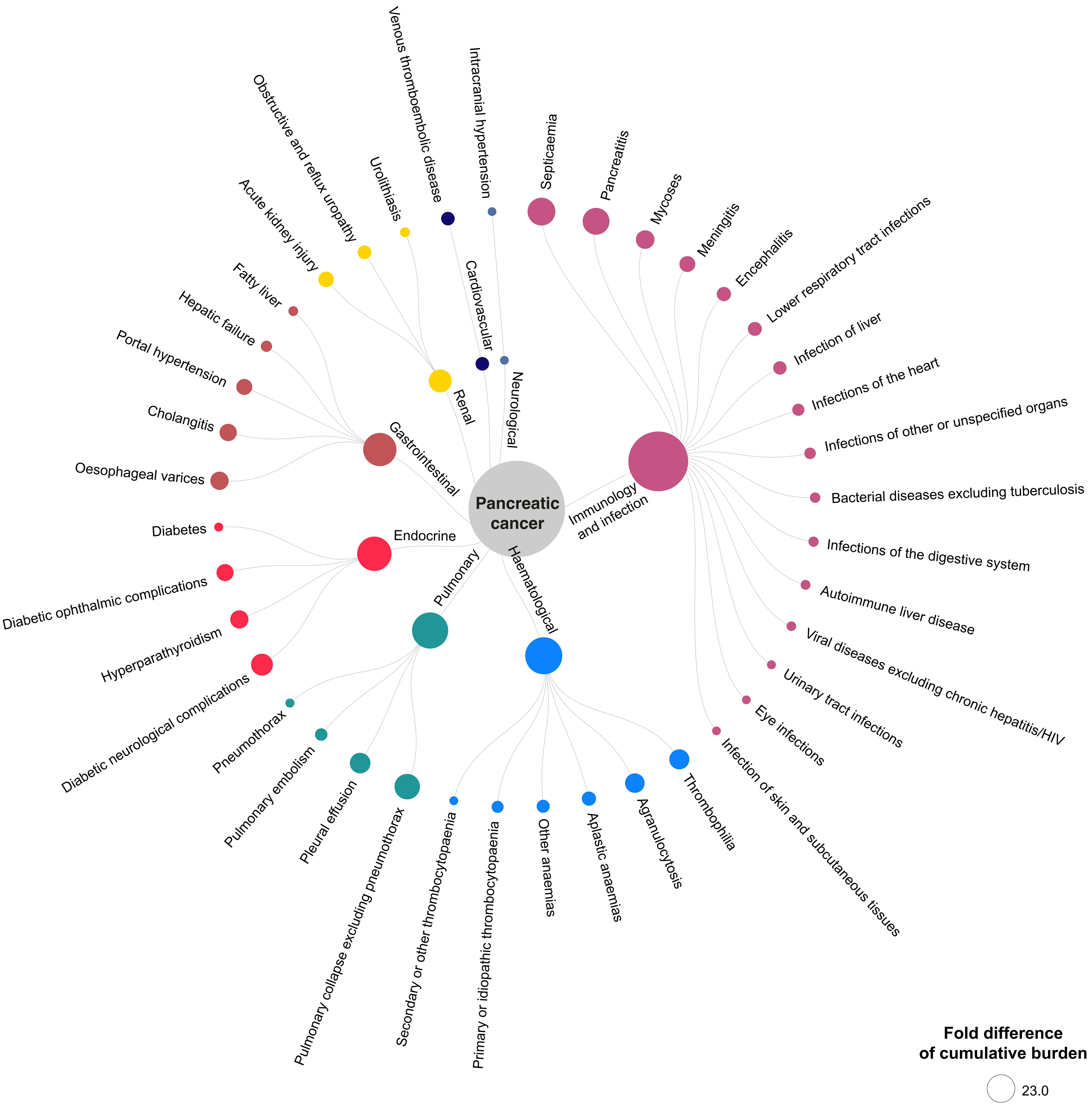

Supplementary Figure 18. Circular dendrogram for small intestine cancer depicting the fold difference of cumulative burden in survivors versus controls at age 60 where conditions with a fold difference of ≥ 2 are shown. The area of the nodes is proportional to the fold-difference of each condition, and the conditions are ranked from the highest to lowest fold difference. Source Data are provided as a Source Data file.

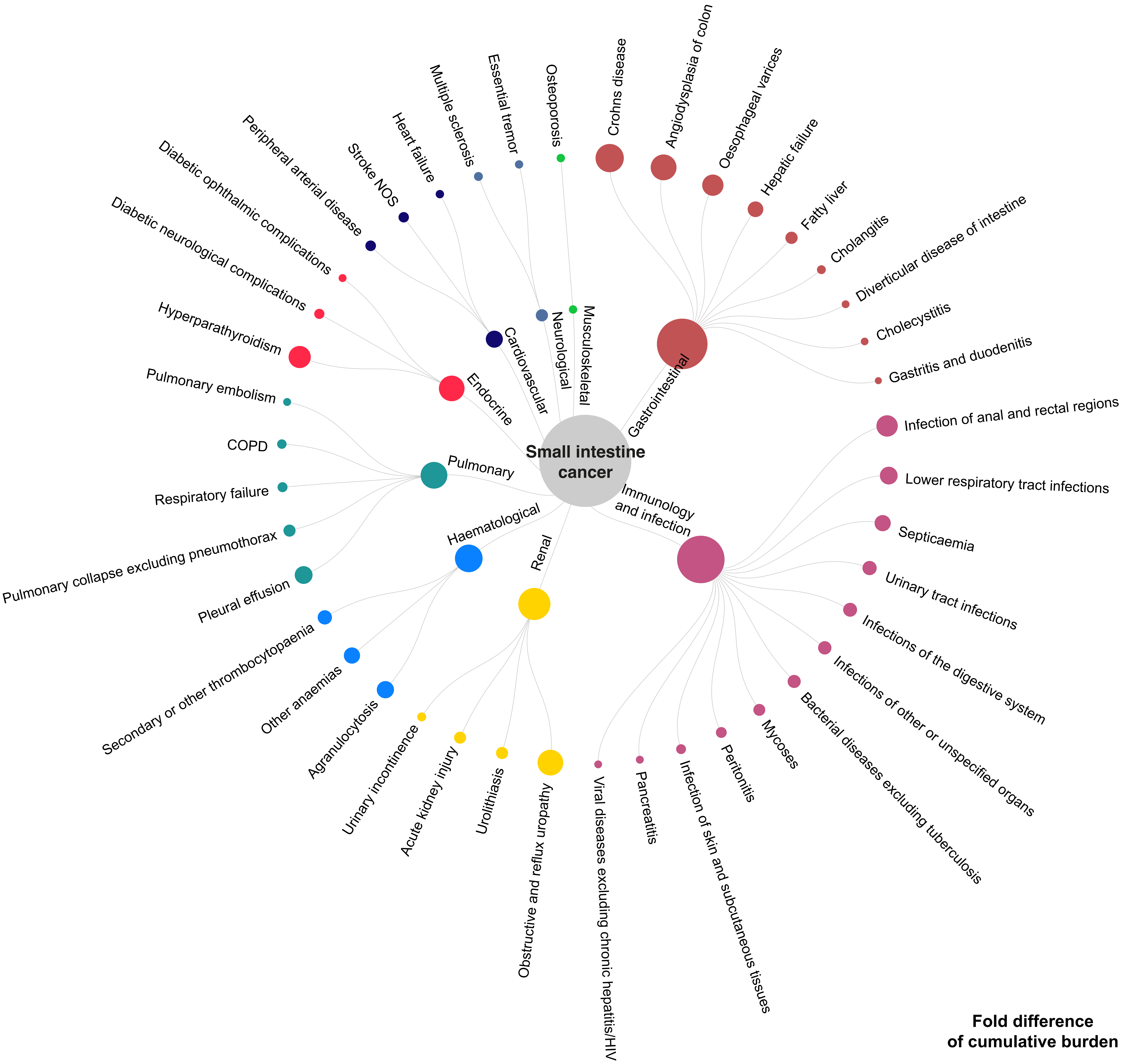

Supplementary Figure 19. Circular dendrogram for spinal cord and nervous system cancer depicting the fold difference of cumulative burden in survivors versus controls at age 60 where conditions with a fold difference of ≥ 2 are shown. The area of the nodes is proportional to the fold-difference of each condition, and the conditions are ranked from the highest to lowest fold difference. Source data are provided as a Source Data file.

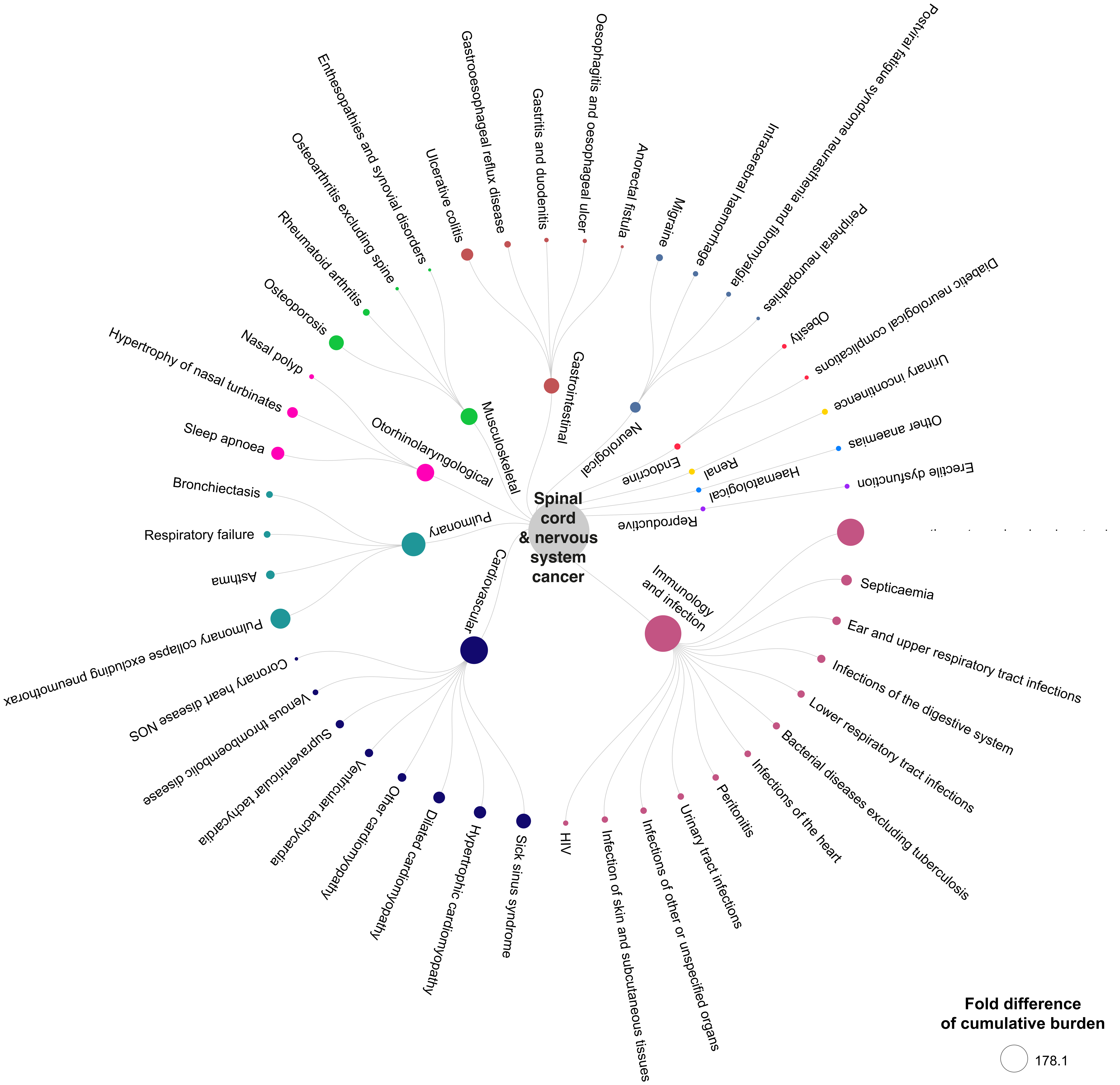

Supplementary Figure 20. Circular dendrogram for stomach cancer depicting the fold difference of cumulative burden in survivors versus controls at age 60 where conditions with a fold difference of ≥ 2 are shown. The area of the nodes is proportional to the fold-difference of each condition, and the conditions are ranked from the highest to lowest fold difference. Source data are provided as a Source Data file.

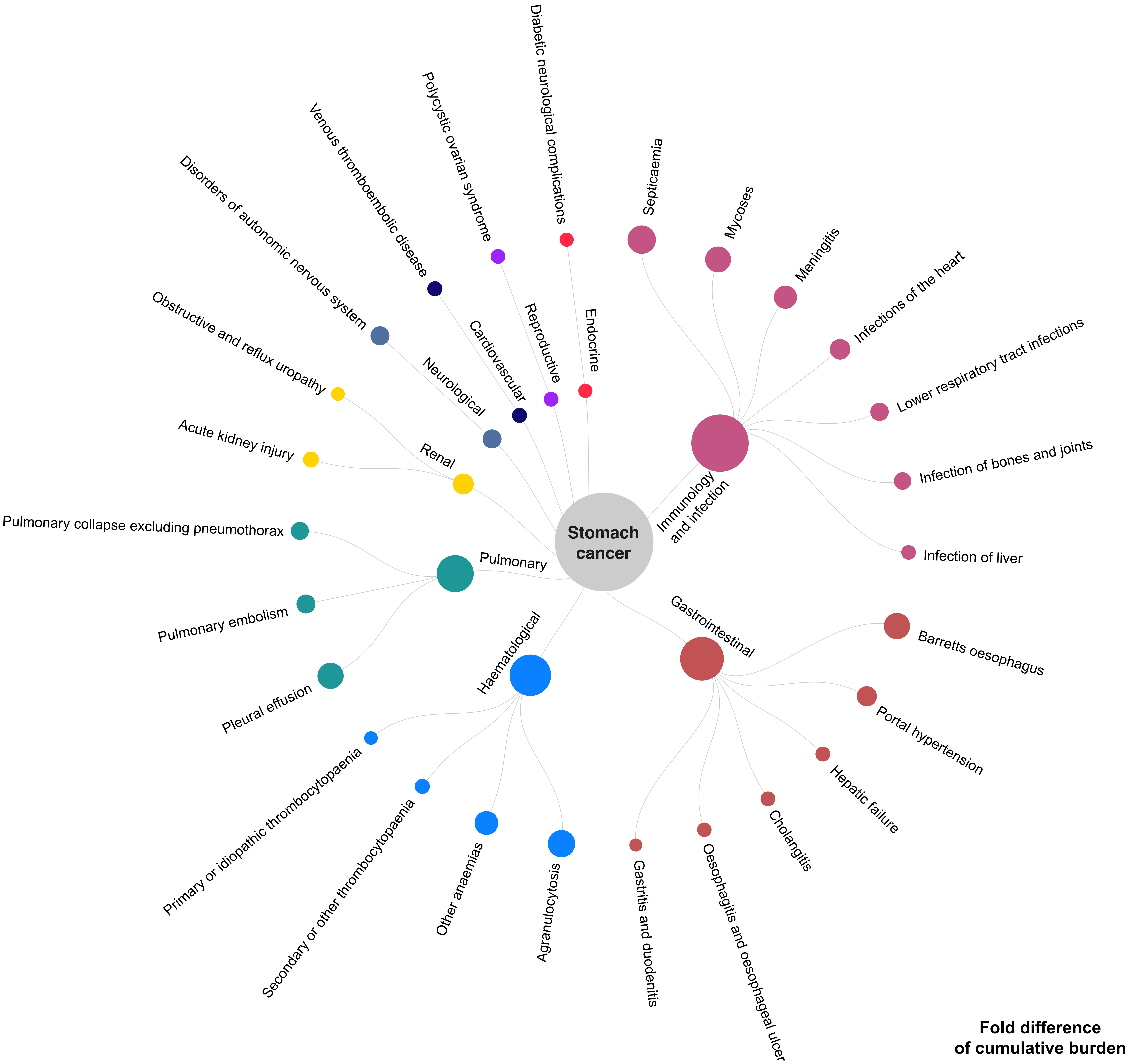

Supplementary Figure 21. Circular dendrogram for testicular cancer depicting the fold difference of cumulative burden in survivors versus controls at age 60 where conditions with a fold difference of ≥ 2 are shown. The area of the nodes is proportional to the fold-difference of each condition, and the conditions are ranked from the highest to lowest fold difference. Source data are provided as a Source Data file.

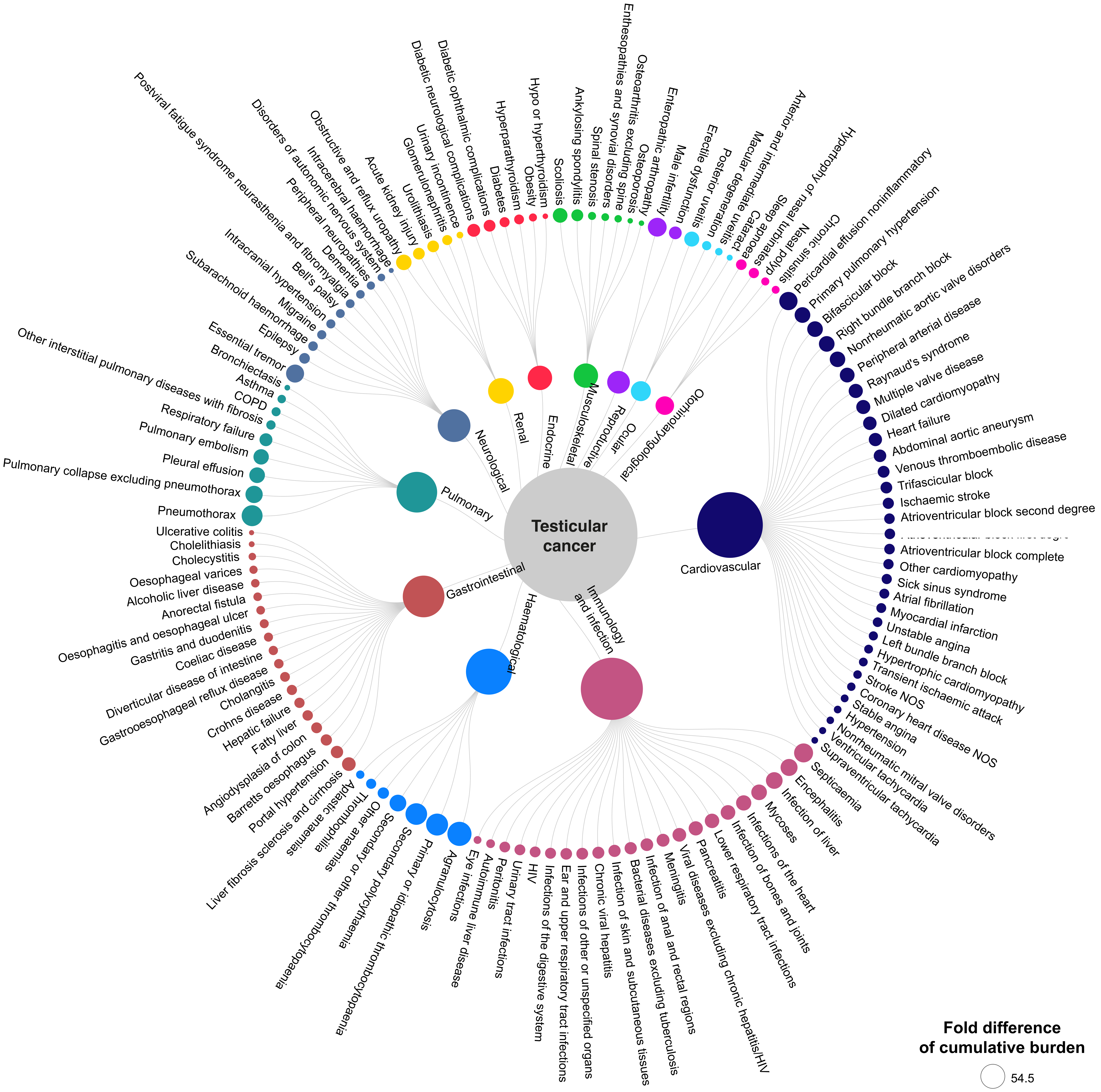

Supplementary Figure 22. Circular dendrogram for thyroid cancer depicting the fold difference of cumulative burden in survivors versus controls at age 60 where conditions with a fold difference of ≥ 2 are shown. The area of the nodes is proportional to the fold-difference of each condition, and the conditions are ranked from the highest to lowest fold difference. Source data are provided as a Source Data file.

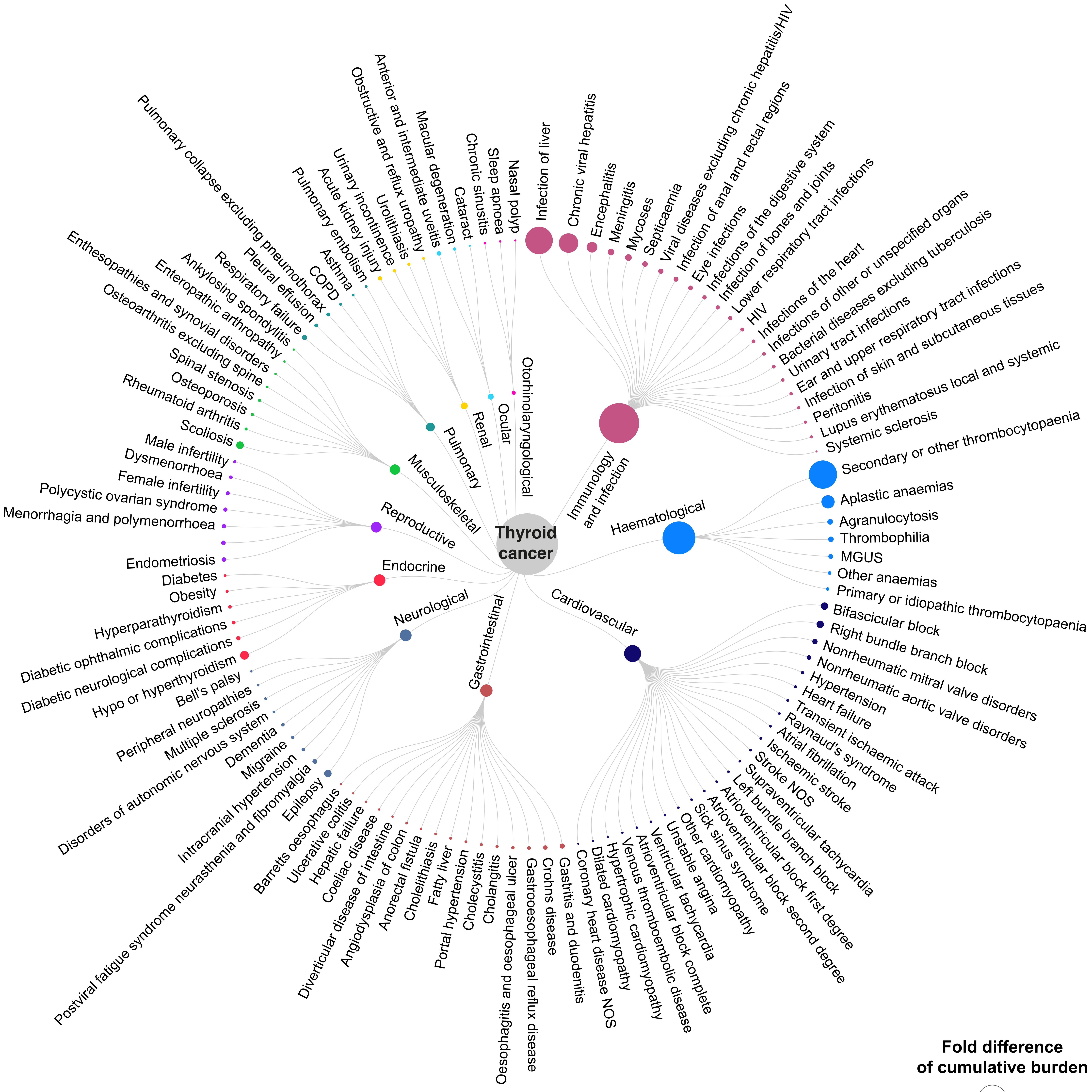

Supplementary Figure 23. Circular dendrogram for uterine cancer depicting the fold difference of cumulative burden in survivors versus controls at age 60 where conditions with a fold difference of ≥ 2 are shown. The area of the nodes is proportional to the fold-difference of each condition, and the conditions are ranked from the highest to lowest fold difference. Source data are provided as a Source Data file.

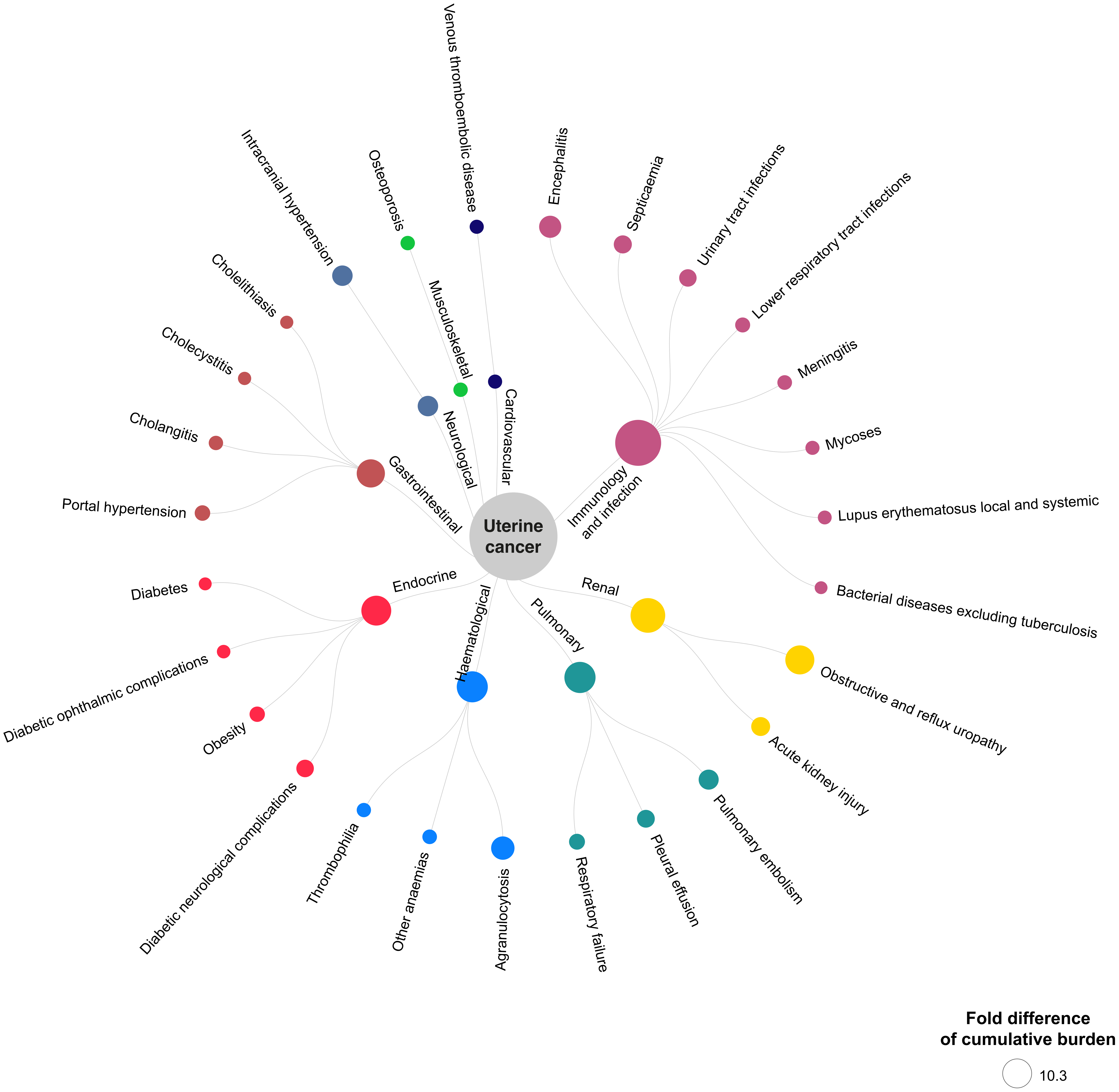

Supplementary Figure 24. Area charts display the cumulative burden of individual cardiovascular conditions according to follow-up time across 26 cancer types. Source data are provided as a Source Data file.

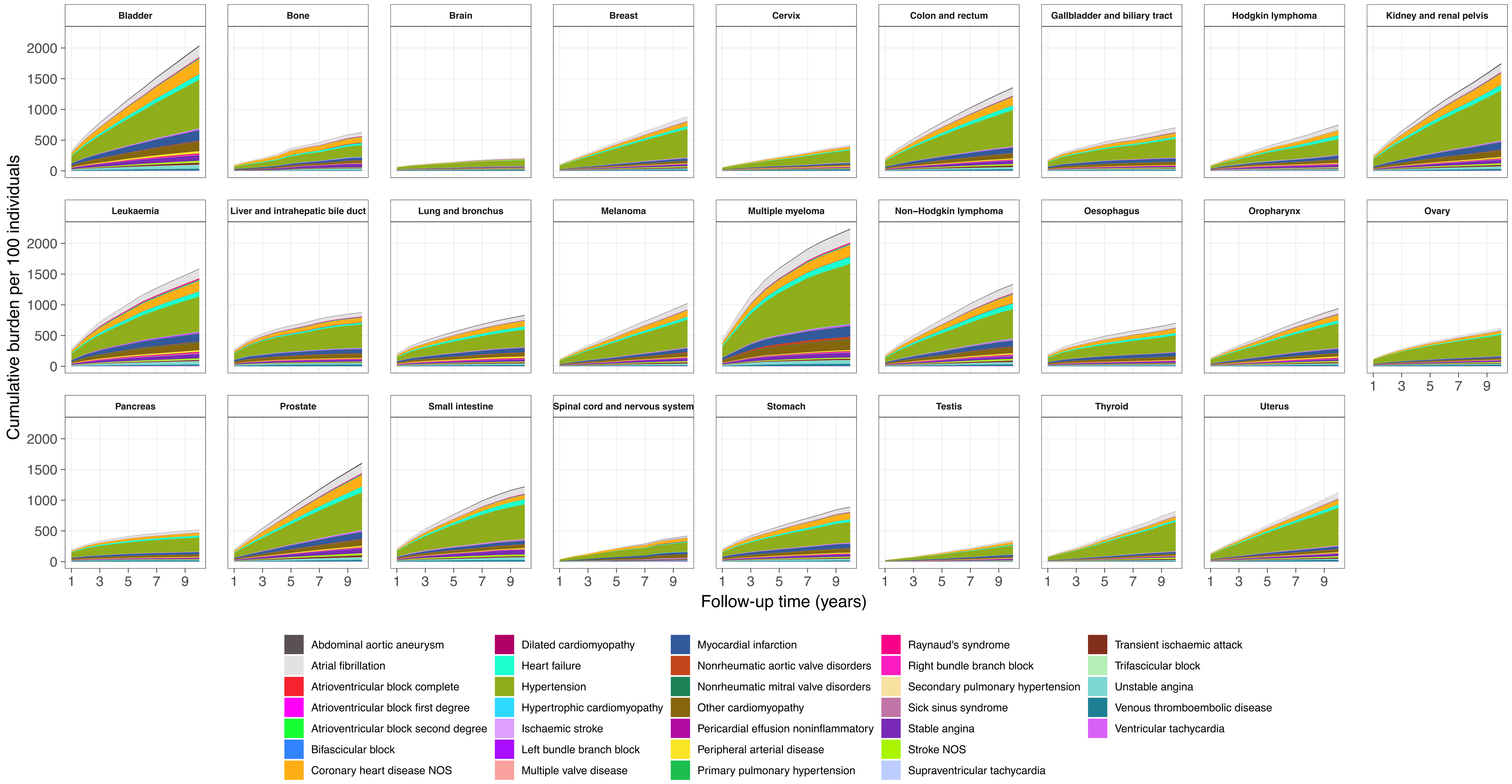

Supplementary Figure 25. Area charts display the cumulative burden of individual gastrointestinal conditions according to follow-up time across 26 cancer types. Source data are provided as a Source Data file.

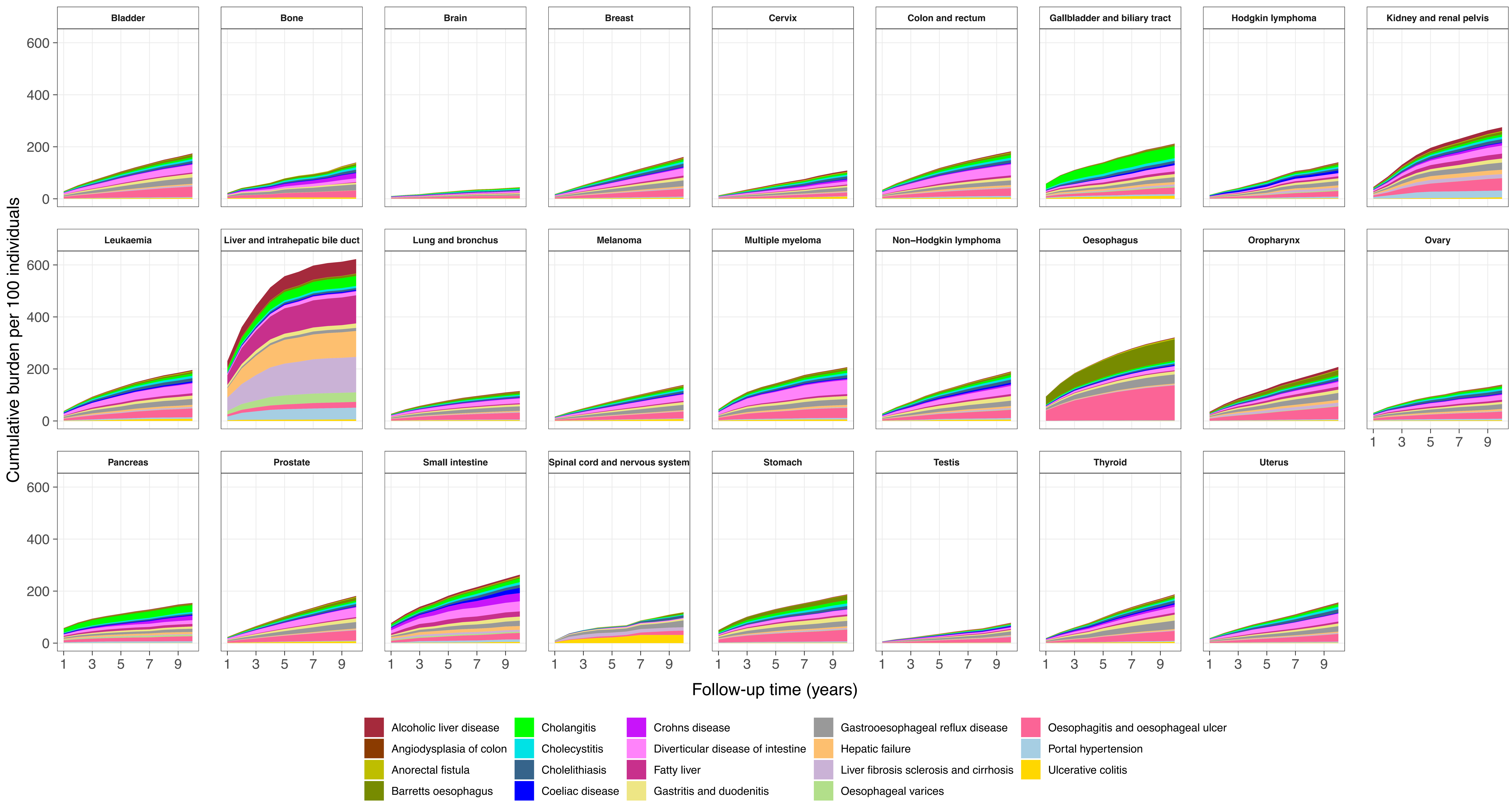

Supplementary Figure 26. Area charts display the cumulative burden of individual immunology and infection conditions according to follow-up time across 26 cancer types. Source data are provided as a Source Data file.

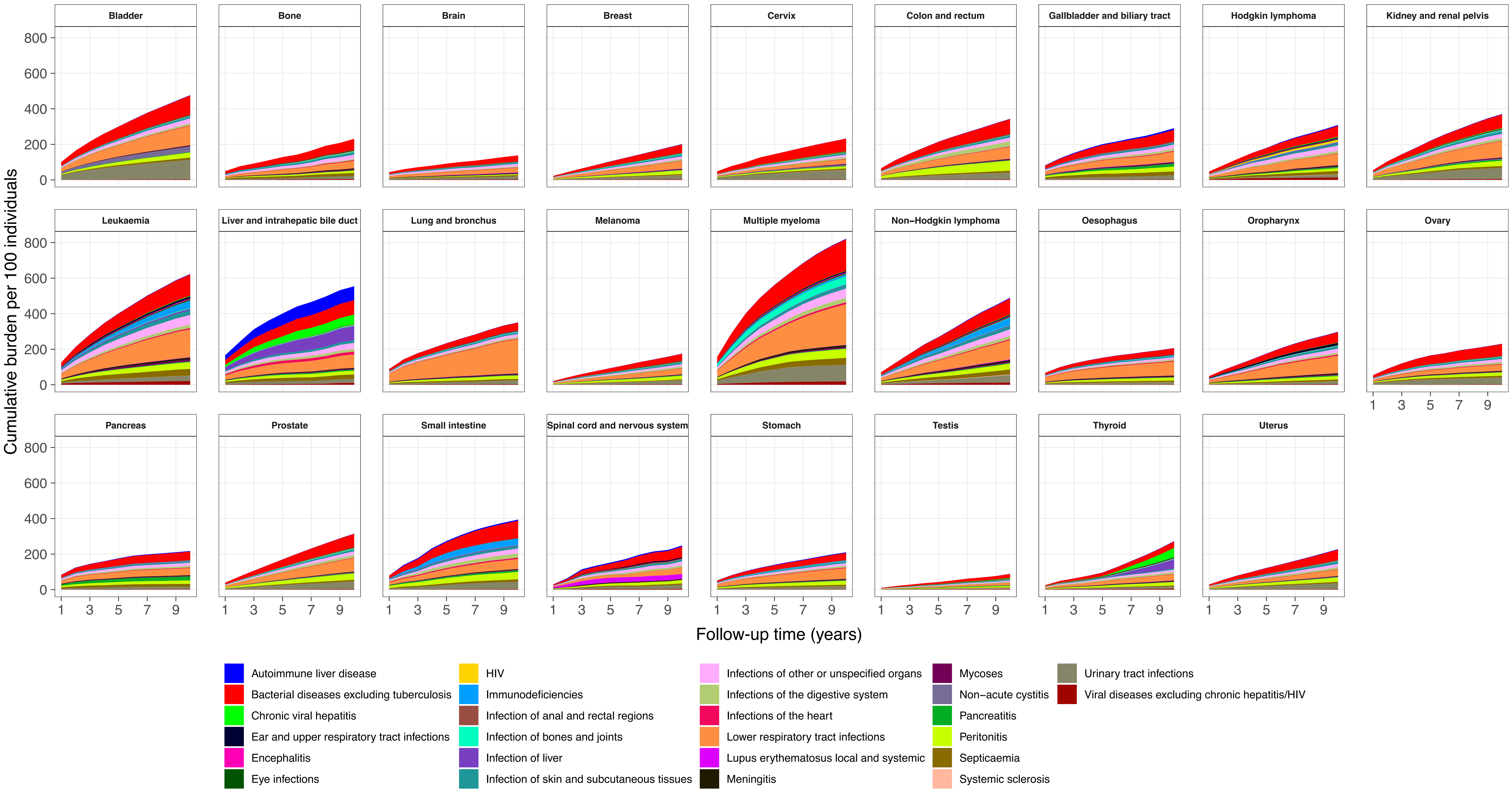

Supplementary Figure 27. Area charts display the cumulative burden of individual musculoskeletal conditions according to follow-up time across 26 cancer types. Source data are provided as a Source Data file.

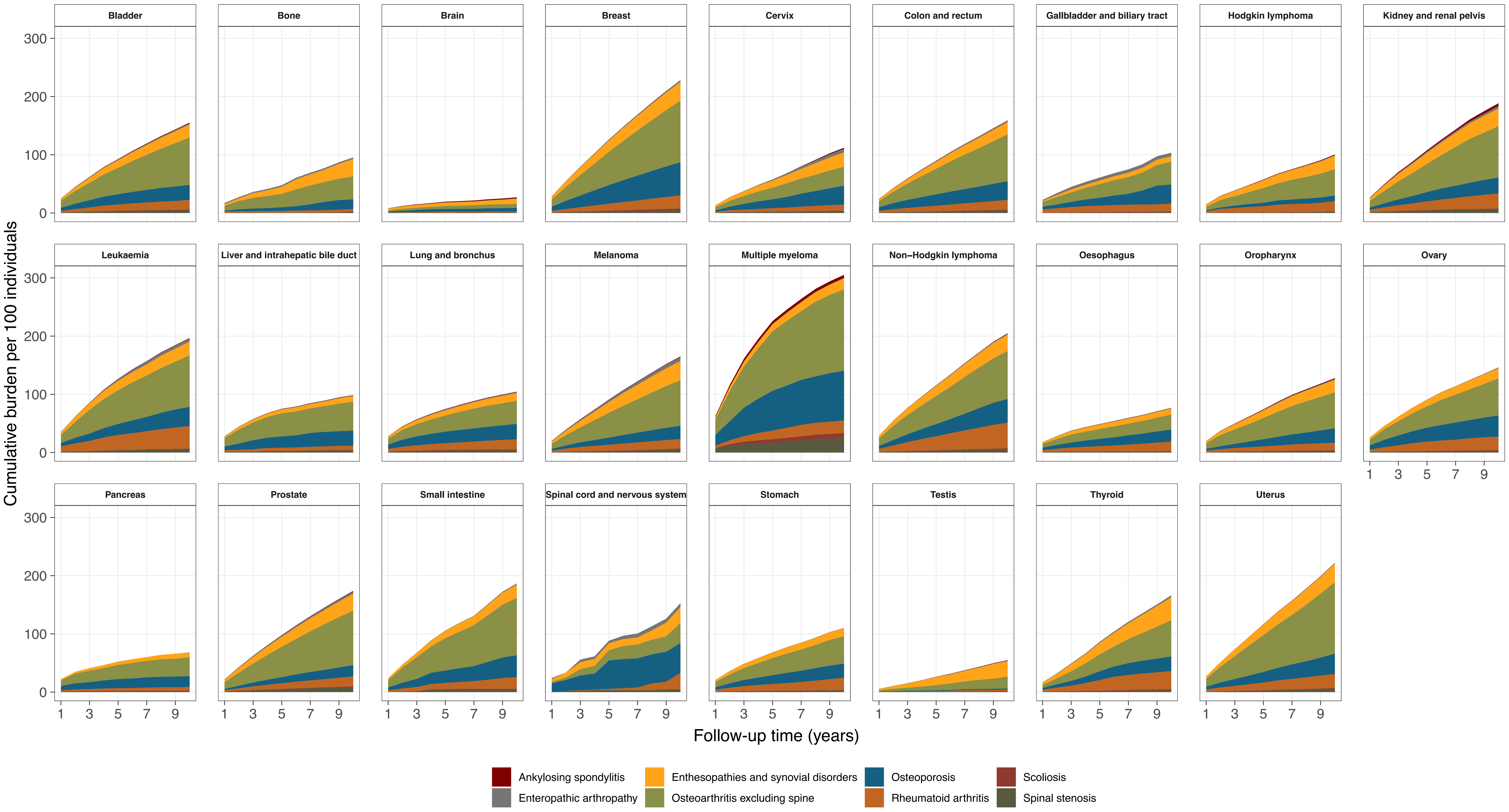

Supplementary Figure 28. Area charts display the cumulative burden of individual neurological conditions according to follow-up time across 26 cancer types. Source data are provided as a Source Data file.

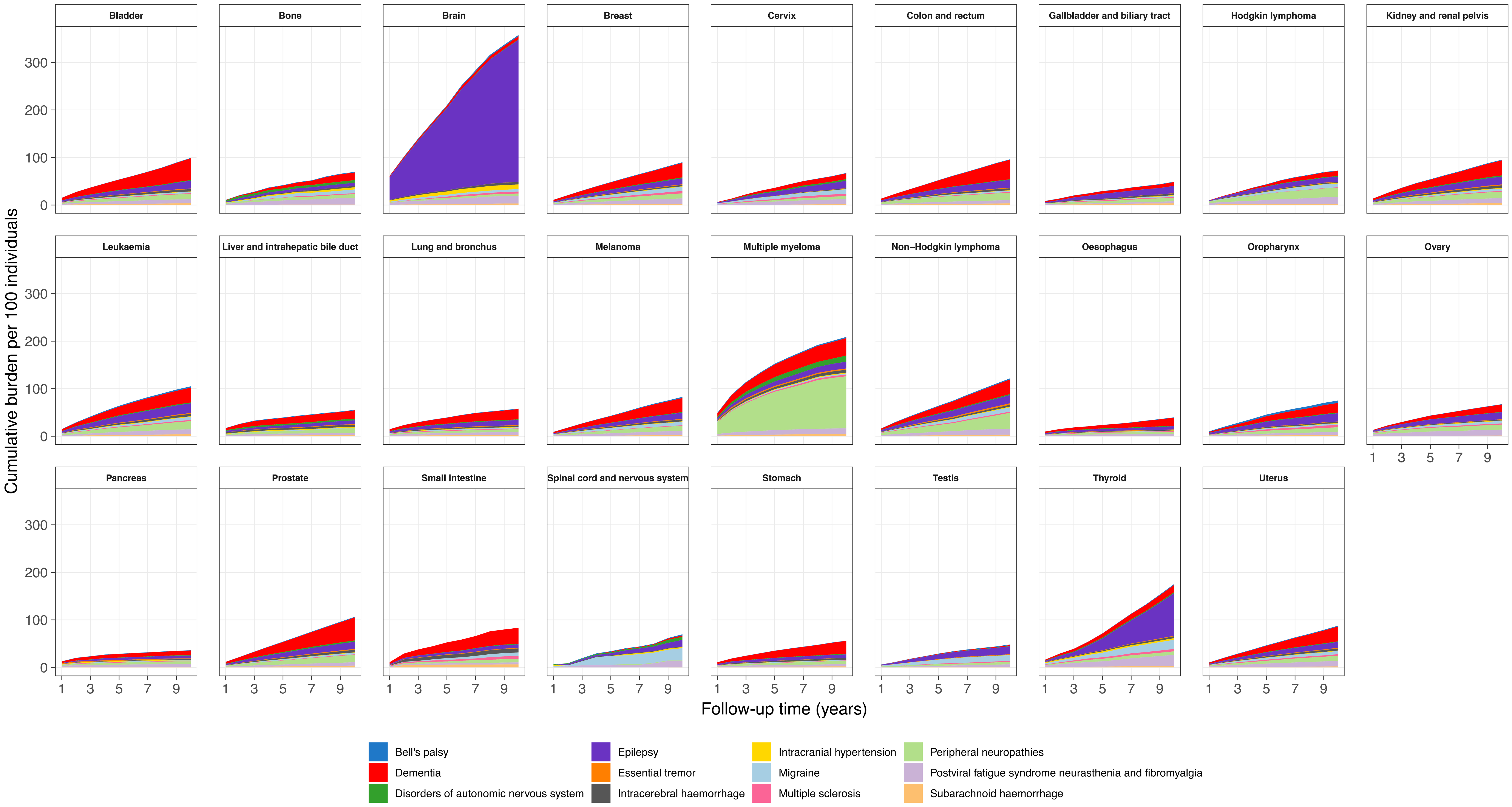

Supplementary Figure 29. Area charts display the cumulative burden of individual ocular conditions according to follow-up time across 26 cancer types. Source data are provided as a Source Data file.

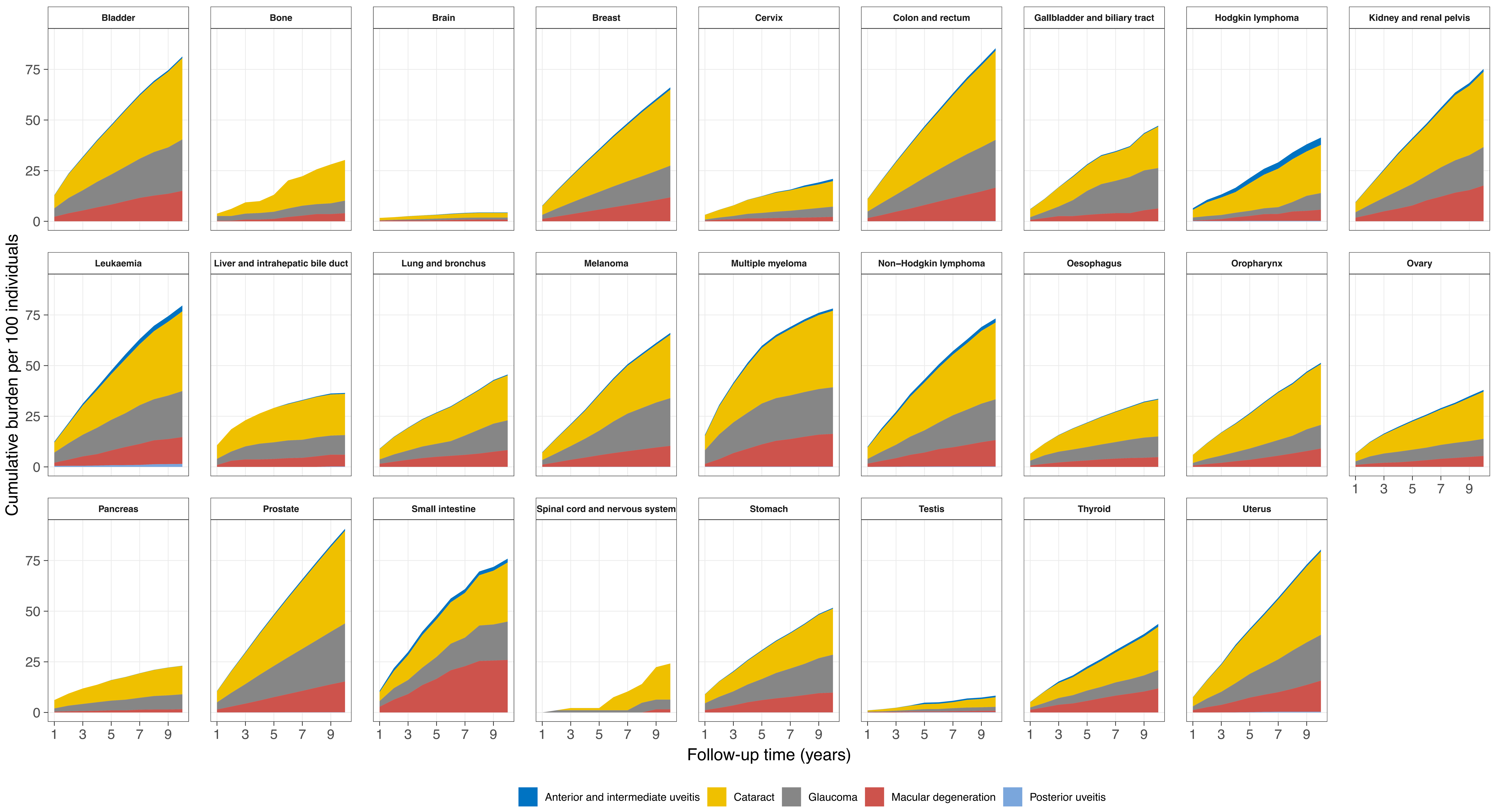

Supplementary Figure 30. Area charts display the cumulative burden of individual otorhinolaryngological conditions according to follow-up time across 26 cancer types. Source data are provided as a Source Data file.

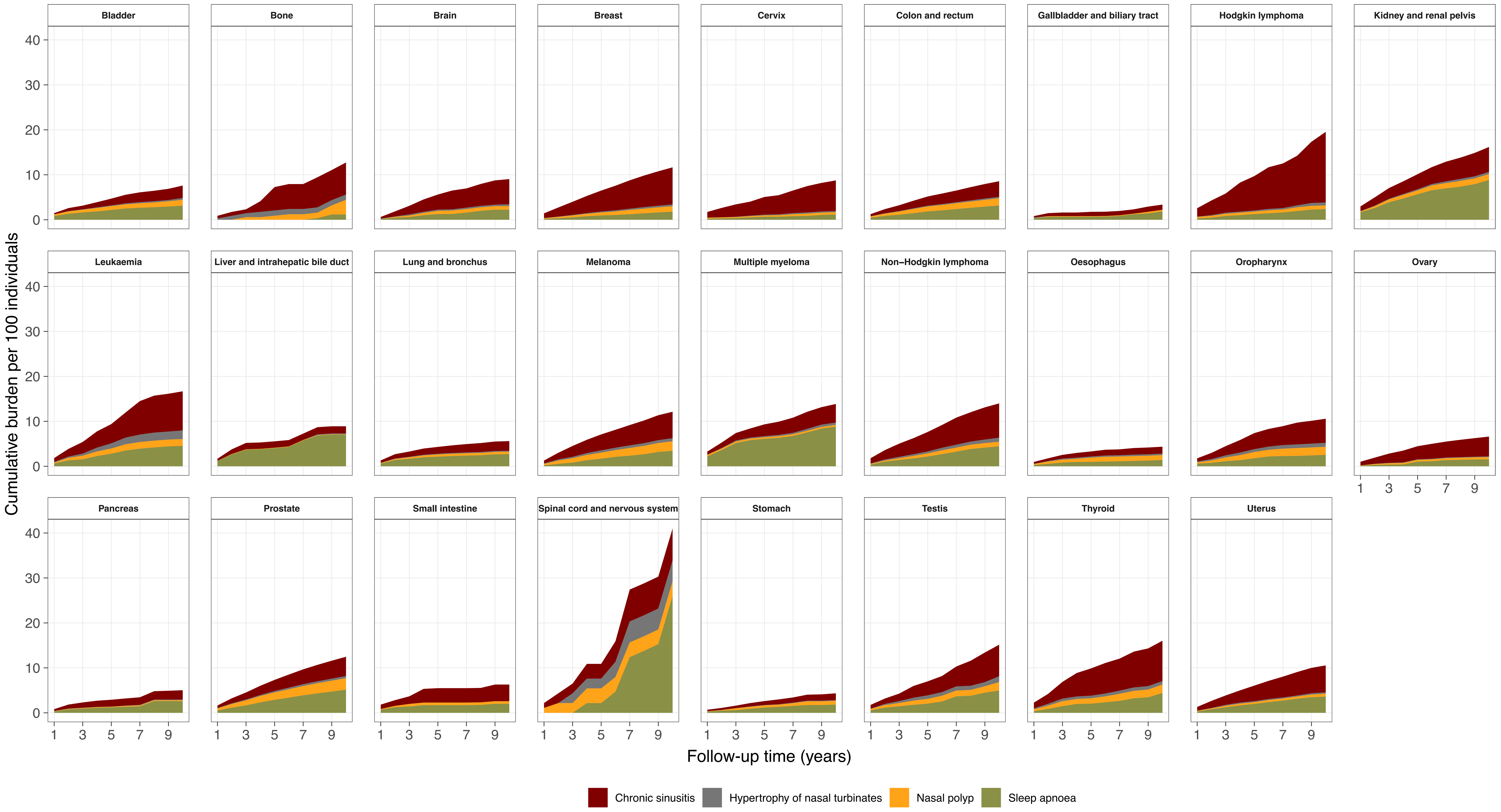

Supplementary Figure 31. Area charts display the cumulative burden of individual pulmonary conditions according to follow-up time across 26 cancer types. Source data are provided as a Source Data file.

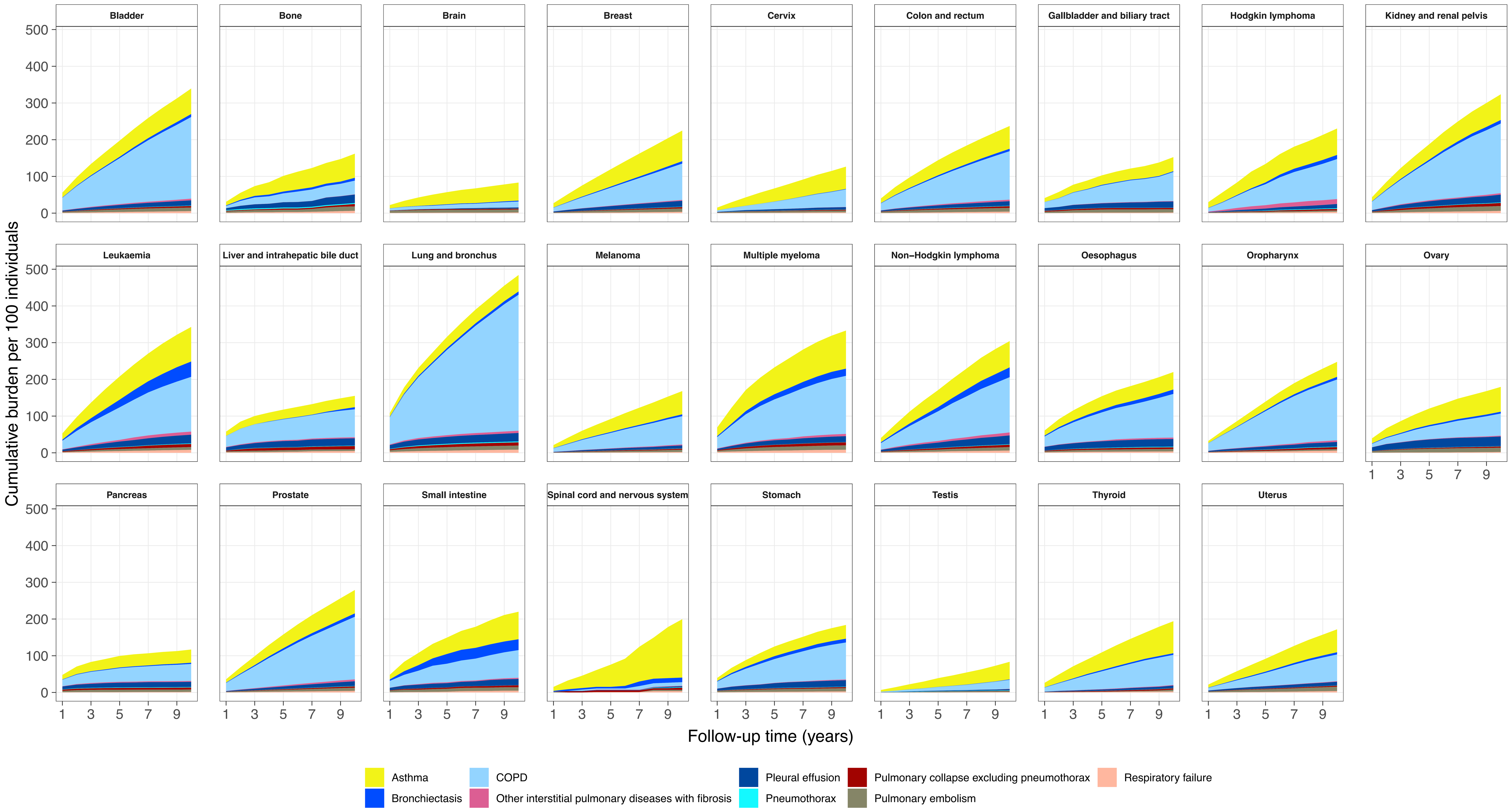

Supplementary Figure 32. Area charts display the cumulative burden of individual renal conditions according to follow-up time across 26 cancer types. Source data are provided as a Source Data file.

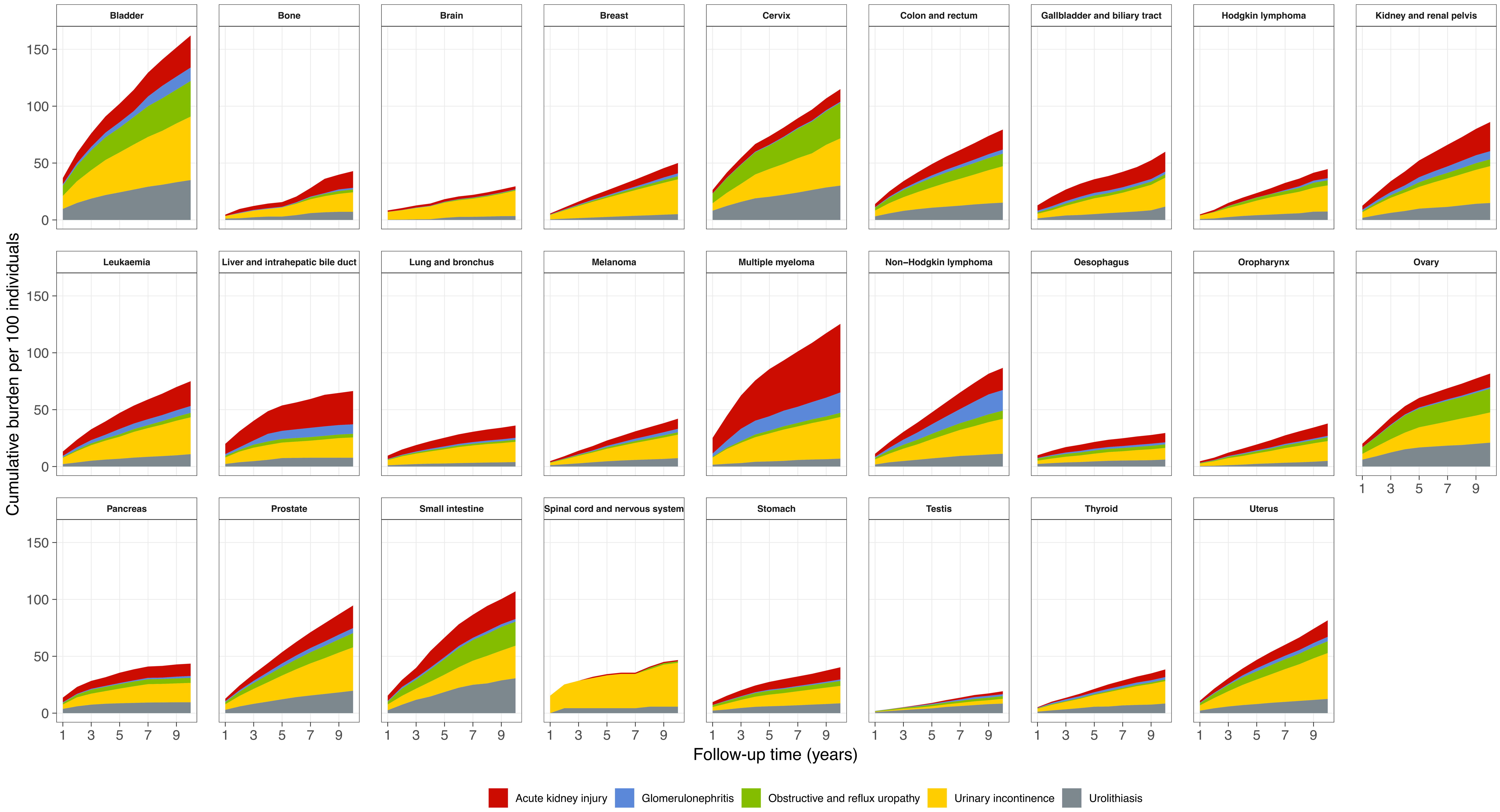

Supplementary Figure 33. Area charts display the cumulative burden of individual reproductive conditions according to follow-up time across 26 cancer types. Source data are provided as a Source Data file.

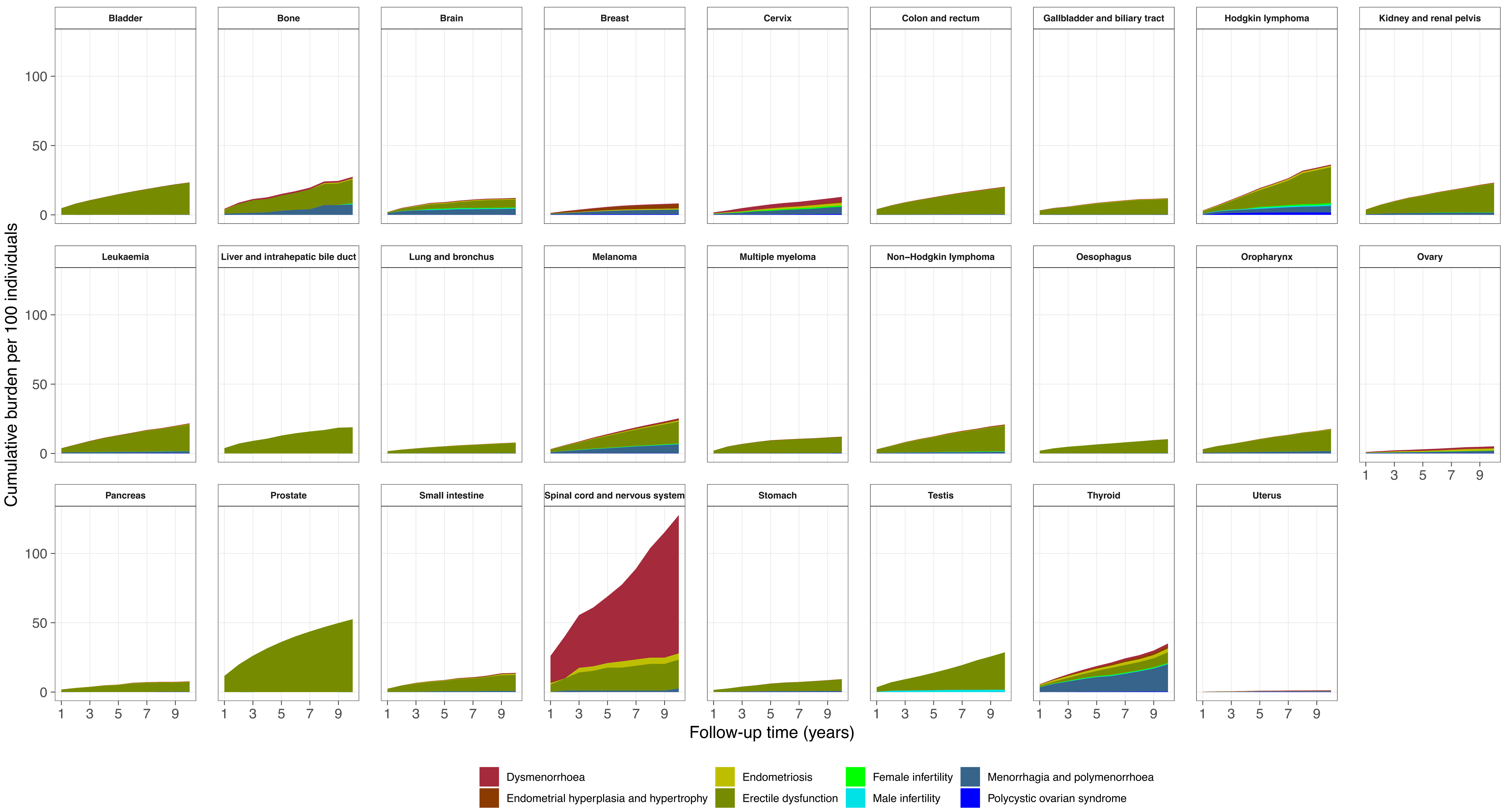

Supplementary Figure 34. Cumulative burden of critical care admission among cancer survivors.

(A) Cumulative burden stratified by primary cancer diagnosis.

(B) Cumulative burden stratified by treatment type.

(C) Cumulative burden stratified by chemotherapy type. Source data are provided as a Source Data file.

A

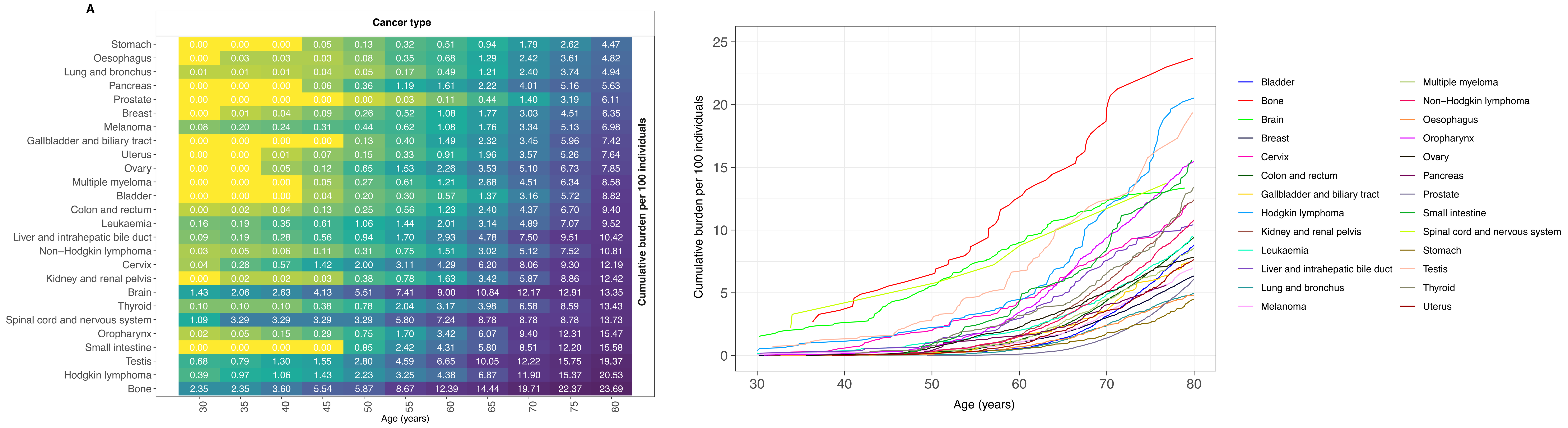

B

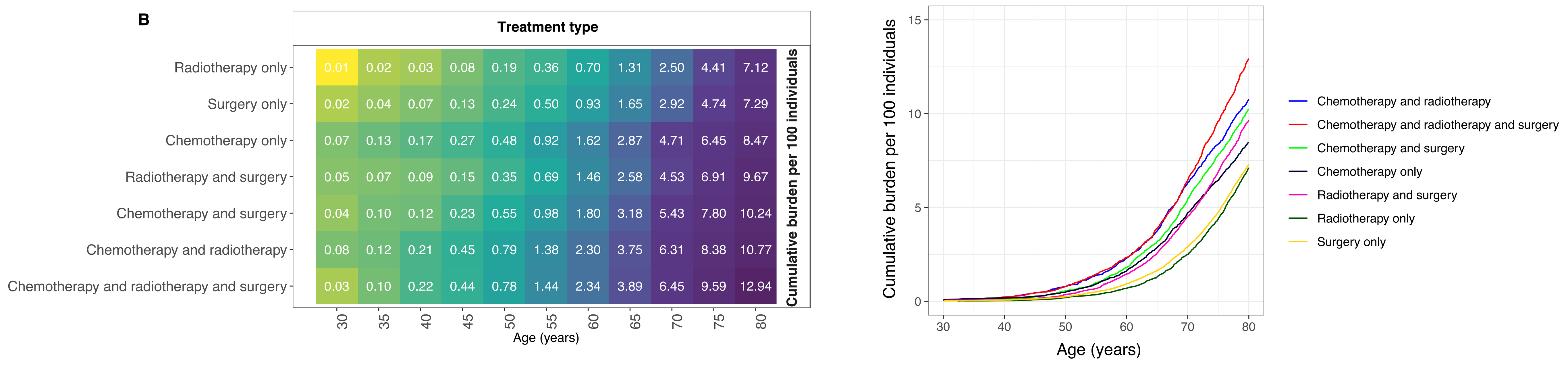

C

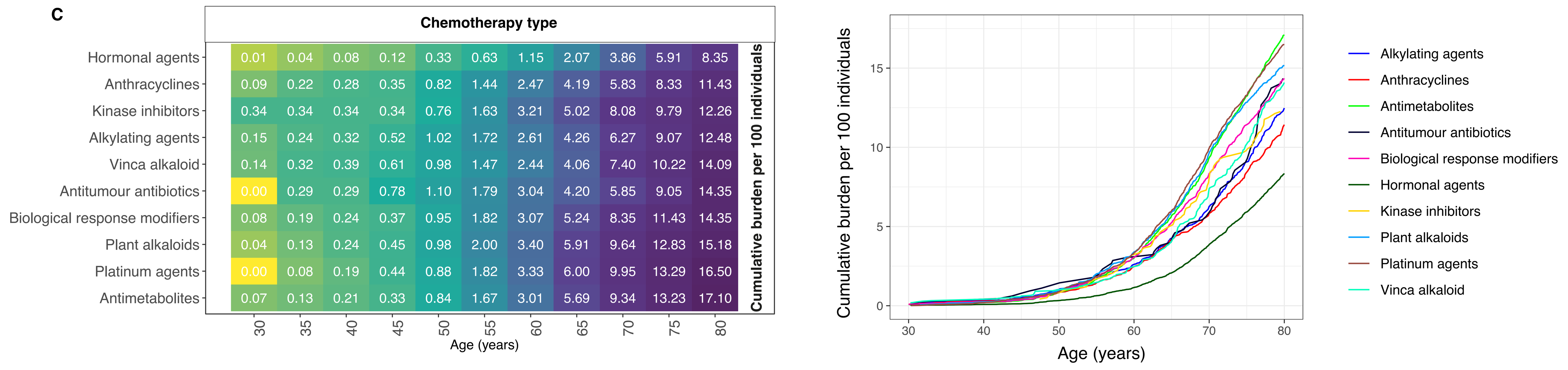

Supplement: Supplementary file 1 — Supplementary Information [file 41467_2023_37231_MOESM1_ESM.pdf]
