# Supplementary material for: Cumulative burden of 144 conditions, critical care hospitalisation and premature mortality across 26 adult cancers
Source: Nat Commun. 2023 Mar 17;14:1484. doi: 10.1038/s41467-023-37231-3 (PMC10023774; doi:10.1038/s41467-023-37231-3)
Supplement: Supplementary file 2 — Description of Additional Supplementary Files [file 41467_2023_37231_MOESM2_ESM.pdf]

Description of Additional Supplementary Files

Supplementary Data 1: Baseline characteristics of cancer survivors and controls.

Supplementary Data 2: List of 144 health conditions grouped by organ systems.

Supplementary Data 3: Cumulative burden of health conditions stratified by indices of multiple deprivation (IMD) for all survivors and controls at 40, 50, 60, 70 and 80 years attained age. Cumulative burden is shown as mean number of events per 100 individuals.

Supplementary Data 4: Cumulative burden of health conditions in cancer survivors stratified by 26 cancer types at 40, 50, 60, 70 and 80 years attained age. Cumulative burden is shown as mean number of events per 100 individuals.

Supplementary Data 5: Fold difference of cumulative burden in survivors versus controls at age 60 for specific cancer types where conditions with a fold difference of ≥ 2 are shown.

Supplementary Data 6: Cumulative burden of health conditions in cancer survivors stratified by 26 cancer types according to follow-up time (1 year to 10 years). Cumulative burden is shown as mean number of events per 100 individuals.

Supplementary Data 7: Cumulative burden of health conditions in cancer survivors stratified by 7 treatment types at 40, 50, 60, 70 and 80 years attained age. Cumulative burden is shown as mean number of events per 100 individuals.

Supplementary Data 8: Cumulative burden of health conditions in cancer survivors stratified by 10 chemotherapy types at 40, 50, 60, 70 and 80 years attained age. Cumulative burden is shown as mean number of events per 100 individuals.

Supplementary Data 9: Cumulative burden of critical care admissions in cancer survivors stratified by 26 cancer types at 5-year age increments. Cumulative burden is shown as mean number of events per 100 individuals.

Supplementary Data 10: Cumulative burden of critical care admissions in cancer survivors stratified by 7 treatment types at 5-year age increments. Cumulative burden is shown as mean number of events per 100 individuals.

Supplementary Data 11: Cumulative burden of critical care admissions in cancer survivors stratified by 10 chemotherapy types at 5-year age increments. Cumulative burden is shown as mean number of events per 100 individuals.

Supplementary Data 12: Logistic regression analysis for the association between treatment type and diagnoses of health conditions among cancer survivors. The Wald test is employed.

Supplementary Data 13: Logistic regression analysis for the association between chemotherapy type and diagnoses of health conditions among cancer survivors. The Wald test is employed.

Supplementary Data 14: Excess years of life lost (YLL) attributable to health conditions among cancer survivors. Excess YLL denotes the difference in years of

life lost between two groups: survivors who developed a health condition compared with survivors who did not develop a health condition.

Supplementary Data 15: Excess years of life lost (YLL) by organ system among cancer survivors. Excess YLL denotes the difference in years of life lost between two groups: survivors who developed a health condition compared with survivors who did not develop a health condition.
