# Supplementary material for: Cumulative burden of 144 conditions, critical care hospitalisation and premature mortality across 26 adult cancers
Source: Nat Commun. 2023 Mar 17;14:1484. doi: 10.1038/s41467-023-37231-3 (PMC10023774; doi:10.1038/s41467-023-37231-3)
Supplement: Supplementary file 4 — Reporting Summary [file 41467_2023_37231_MOESM4_ESM.pdf]

nature portfolio

Corresponding author(s): Alvina Lai

Last updated by author(s): Mar 2, 2023

Reporting Summary

Nature Portfolio wishes to improve the reproducibility of the work that we publish. This form provides structure for consistency and transparency in reporting. For further information on Nature Portfolio policies, see our Editorial Policies and the Editorial Policy Checklist.

Statistics

For all statistical analyses, confirm that the following items are present in the figure legend, table legend, main text, or Methods section.

n/a	Confirmed	
☐	☒	The exact sample size (*n*) for each experimental group/condition, given as a discrete number and unit of measurement
☐	☒	A statement on whether measurements were taken from distinct samples or whether the same sample was measured repeatedly
☐	☒	The statistical test(s) used AND whether they are one- or two-sided<br>*Only common tests should be described solely by name; describe more complex techniques in the Methods section.*
☐	☒	A description of all covariates tested
☒	☐	A description of any assumptions or corrections, such as tests of normality and adjustment for multiple comparisons
☐	☒	A full description of the statistical parameters including central tendency (e.g. means) or other basic estimates (e.g. regression coefficient) AND variation (e.g. standard deviation) or associated estimates of uncertainty (e.g. confidence intervals)
☐	☒	For null hypothesis testing, the test statistic (e.g. *F*, *t*, *r*) with confidence intervals, effect sizes, degrees of freedom and *P* value noted<br>*Give P values as exact values whenever suitable.*
☒	☐	For Bayesian analysis, information on the choice of priors and Markov chain Monte Carlo settings
☒	☐	For hierarchical and complex designs, identification of the appropriate level for tests and full reporting of outcomes
☒	☐	Estimates of effect sizes (e.g. Cohen's *d*, Pearson's *r*), indicating how they were calculated

Our web collection on statistics for biologists contains articles on many of the points above.

Software and code

Policy information about availability of computer code

Data collection	No software was used for data collection.
Data analysis	All analyses were performed using R Studio (v3.6.3) within an airlocked research environment using analysis codes retrieved from this website (https://sites.ualberta.ca/~yyasui/software.html). The following packages were used: tidyverse (v1.3.2), tableone (v0.13.0), etm (v1.1.1), mstate (v0.3.2), cmprsk (v2.2.11), lillies (v0.2.9), reshape (v0.8.9), splines (v0.4.5), survival (v3.3), survminer (v0.4.9), matchit (v4.3.4), DataCombine (v0.2.21) and data.table (v1.14.6).

For manuscripts utilizing custom algorithms or software that are central to the research but not yet described in published literature, software must be made available to editors and reviewers. We strongly encourage code deposition in a community repository (e.g. GitHub). See the Nature Portfolio guidelines for submitting code & software for further information.

Data

Policy information about availability of data

All manuscripts must include a data availability statement. This statement should provide the following information, where applicable:

- Accession codes, unique identifiers, or web links for publicly available datasets
- A description of any restrictions on data availability
- For clinical datasets or third party data, please ensure that the statement adheres to our policy

This study employs patient data in England collected as part of their care and support. The data is subject to controlled access and reason for controlled access is as follows: since electronic health records are classified as sensitive data by the UK Data Protection Act, information governance restrictions are in place to protect

patient confidentiality and prevent data sharing in public repositories. Data access is conditioned on successful ethics application to the Medicines and Healthcare products Regulatory Agency and assessment by the Independent Scientific Advisory Committee. All summarised data and results are provided as a Source Data file and further enquiries can be directed to the lead author Wai Hoong Chang (wai.chang@ucl.ac.uk) who will aim to respond to requests within 2 weeks.

Human research participants

Policy information about studies involving human research participants and Sex and Gender in Research.

Reporting on sex and gender	Findings apply to both sexes. However, certain cancer types are sex-specific (e.g., prostate and ovarian cancers). Regression analyses were adjusted for sex. Sex was included as one of the variables for propensity score matching.
Population characteristics	We identified 243,767 adults with an incident site-specific cancer diagnosis and who survived for at least 1 year. Cancer survivors were matched to 506,892 controls. The mean ages for cancer survivors (66.7 years) and matched controls (66.6 years) were comparable. 48.3% and 49.1% of cancer survivors and controls were men, respectively.
Recruitment	This study employs electronic health records collected as part of routine clinical care. No patient recruitment was performed.
Ethics oversight	Ethics approval was obtained from the Medicines and Healthcare products Regulatory Agency (19 222).

Note that full information on the approval of the study protocol must also be provided in the manuscript.

Field-specific reporting

Please select the one below that is the best fit for your research. If you are not sure, read the appropriate sections before making your selection.

☒ Life sciences ☐ Behavioural & social sciences ☐ Ecological, evolutionary & environmental sciences

For a reference copy of the document with all sections, see nature.com/documents/nr-reporting-summary-flat.pdf

Life sciences study design

All studies must disclose on these points even when the disclosure is negative.

Sample size	Cancer survivors were grouped according to 26 site-specific diagnostic categories: bladder (9,652 individuals), bone (343), brain (1,614), breast (57,365), cervix (2,547), colon and rectum (35,054), gallbladder and biliary tract (744), Hodgkin lymphoma (1,286), kidney and renal pelvis (6,374), leukaemia (5,747), liver and intrahepatic bile duct (1,063), lung and bronchus (13,993), melanoma (12,278), multiple myeloma (3,765), non-Hodgkin lymphoma (10,163), oesophagus (3,998), oropharynx (5,491), ovary (5,816), pancreas (1,696), prostate (47,614), small intestine (710), spinal cord and nervous system (92), stomach (3,764), testis (1,921), thyroid (1,937) and uterus (8,740). Sample sizes for cancer survivors were determined based on the presence of a specific cancer diagnostic code in the patient's medical history. Cancer survivors were matched to 506,892 controls. Covariates used for propensity score matching were year of birth, sex and socioeconomic deprivation status.
Data exclusions	Only incident primary site-specific cancer cases were included.
Replication	As this is an observational epidemiological study using a population-based cohort, experimental replication is not applicable.
Randomization	This is an observational epidemiological study. Thus randomisation is not applicable. However, we have taken measures to ensure that cases and controls were comparable using propensity score matching.
Blinding	This is an observational epidemiological study thus blinding is not applicable.

Reporting for specific materials, systems and methods

We require information from authors about some types of materials, experimental systems and methods used in many studies. Here, indicate whether each material, system or method listed is relevant to your study. If you are not sure if a list item applies to your research, read the appropriate section before selecting a response.

Materials & experimental systems

n/a	Involved in the study
☒	☐ Antibodies
☒	☐ Eukaryotic cell lines
☒	☐ Palaeontology and archaeology
☒	☐ Animals and other organisms
☒	☐ Clinical data
☒	☐ Dual use research of concern

Methods

n/a	Involved in the study
☒	☐ ChIP-seq
☒	☐ Flow cytometry
☒	☐ MRI-based neuroimaging
